# Response Facilitation in Dementia Care: Exploring Engagement Through Social Contexts: A Qualitative Study in Dutch Nursing Homes

**DOI:** 10.3390/healthcare14040539

**Published:** 2026-02-22

**Authors:** Coosje Hammink, Nienke Moor, Masi Mohammadi

**Affiliations:** 1 Department of Built Environment, HAN University of Applied Sciences, 6826 CC Arnhem, The Netherlands; nienke.moor@han.nl (N.M.); m.mohammadi@tue.nl (M.M.); 2 Department of Built Environment, Technische Universiteit Eindhoven, 5600 MB Eindhoven, The Netherlands

**Keywords:** dementia care, social cognitive theory, response facilitation, social engagement, nursing homes, recreational activities

## Abstract

**Highlights:**

**What are the main findings?**

- Group-based recreational activities seem to provide the strongest conditions for response facilitation, leading to more behavioural and physiological signs of engagement among residents with dementia.
- Observational and physiological data frequently diverge, showing that residents may display cognitive or emotional engagement even when not outwardly participating.

**What is the implication of the main finding?**

- The heightened attention seen in group activities suggests a need to understand why these settings seem to be more effective and to translate their social, spatial, and facilitative elements into everyday care contexts to invite broader engagement.
- Reliance on behavioural observation alone may underestimate residents’ internal engagement; care practices and evaluations could incorporate multimodal indicators to better understand and support meaningful participation.

**Abstract:**

Background/Objectives: Dementia-related cognitive impairments and staffing shortages in nursing homes challenge the possibilities for individually tailored recreational activities, raising the question of how the physical and social environment might be leveraged to stimulate engagement through response facilitation, a form of vicarious incentive motivation grounded in Social Cognitive Theory. This study examines in which social contexts observing others’ recreational activities can effectively engage residents with moderate to advanced dementia. Methods: A qualitative, scenario-based multiple case study was conducted in four nursing homes (*n* = 21), using fly-on-the-wall observations, narratives, and three experimentally embedded social contexts (individual, dyadic, group) around familiar leisure activities. Behavioural engagement, mood, and agitation were assessed with validated observational scales (e.g., OERS and MEDLO), complemented by wearable sensor data (HR/PR, HRV/PRV, SCL, and temperature) and video for contextualised interpretation. Results: Across scenarios, non-participating residents showed limited behavioural responses in individual and dyadic settings, while group activities more frequently elicited both observable engagement and physiological markers consistent with attention or cognitive engagement. Observational and physiological data frequently diverged, which may indicate cognitive or emotional engagement even when overt participation or affect remained minimal or appeared negative. Conclusions: Response facilitation appears most likely in structured group activities if supported by explicit social scaffolding, rather than in individual or dyadic constellations alone. Reliance on behavioural observation or environmental design in isolation risks underestimating engagement; multimodal, context-sensitive approaches are recommended to better harness social mechanisms for meaningful participation in dementia care. Future research should integrate contextual factors with physiological measurements and observations as well as further explore patterns of inactivity to distinguish disengagement from subtle forms of cognitive engagement.

## 1. Introduction

The increasing number of older adults with dementia living in nursing homes, in combination with the shortage of care personnel, requires novel approaches to maintain their well-being. Structured care routines and organisational rhythms significantly shape the older adults with dementia’s daily activities [1]. Stimulation-based interventions, including reminiscence therapy, social engagement, and creative hobbies, are frequently employed to support cognitive, physical, and emotional well-being [2,3,4].

These interventions not only depend on limited staff resources but also tend to be implemented in a one-on-one fashion due to the highly person-specific nature of dementia care [5]. While personalised approaches are valuable, they potentially overlook opportunities for broader engagement through social learning mechanisms. However, research looking into these social learning mechanisms has not focused on this specific target group. Given increasing personnel shortages, caregivers must prioritise essential care tasks such as dressing, bathing, and feeding [6] while simultaneously trying to provide individualised recreational activities that are rarely perceived as extending benefits beyond the directly involved resident [7,8]. This individualised approach fails to capitalise on potential ‘ripple effects’ where one resident’s engagement might stimulate interest and participation among others through observational processes [9,10], a phenomenon that could perhaps expand the reach of limited staff resources.

Public health strategies often rely on behaviour change models to foster well-being [11]. Social Cognitive Theory (SCT) provides a useful framework for examining how environmental factors influence behaviour [12,13,14]. SCT underscores how environmental cues act as external motivators that can shape behaviour change. This aligns with stimulation-based interventions (i.e., for older adults with dementia), which leverage environmental and sensory stimuli to engage individuals, particularly by activating cognitive and emotional processes indirectly through the environment [15]. SCT posits that behaviour is shaped by interactions between personal factors, environmental influences, and cognitive processes, such as emotion, memory, and expectation [15,16]. Social Cognitive Theory describes several pathways by which environmental cues can lead to behaviour change, including influencing cognitive processes such as memory and expectation, emotional responses, and social learning mechanisms. In the context of dementia, traditional motivation-based behaviour change strategies, such as self-regulation and goal setting, become less effective due to impairments in memory, executive function, and metacognition [17]. Consequently, identifying alternative mechanisms that leverage external social or physical stimuli to encourage engagement becomes essential.

Prior research using a systematic literature review in combination with expert interviews [18] has identified incentive motivation as a promising mechanism for behaviour change in individuals with moderate to severe dementia. Incentive motivation refers to the process by which external cues in the social or physical environment shape motivation and subsequent behaviours. One specific sub-mechanism of incentive motivation, response facilitation, involves observing others engaging in a behaviour, leading to an increased likelihood of adopting that behaviour oneself [19]. This study investigates the role of response facilitation in encouraging participation in recreational activities among nursing home residents with dementia.

## 2. Theoretical Framework

### 2.1. Social Cognitive Theory and Incentive Motivation

Social Cognitive Theory (SCT) highlights the reciprocal relationship between personal, environmental, and behavioural factors, emphasising cognitive processes as key mediators of behaviour change [12,15,20]. Traditional SCT mechanisms, such as self-efficacy, self-regulation, and outcome expectations, assume an individual’s ability to consciously influence their own motivation and actions [20]. However, dementia-related declines in memory, metacognition, and executive function diminish the applicability of such mechanisms [17,21]. For individuals with moderate to advanced dementia, behaviour change strategies must rely less on these self-directed cognitive processes and more on externally driven mechanisms, of which incentive motivation is an example.

Incentive motivation refers to the process by which external factors, such as rewards, social norms, or environmental stimuli, trigger and sustain behaviour [15]. This mechanism encompasses three primary pathways: (1) direct incentive motivation, where an individual’s own experience with rewards or consequences influences behaviour; (2) vicarious incentive motivation, where observing others experience positive or negative outcomes affects one’s likelihood of engaging in a behaviour; and (3) self-monitored incentive motivation, which relies on personal reflection and self-regulation [22]. Given that dementia negatively impacts self-monitoring and long-term memory recall, direct and vicarious incentive motivation become particularly relevant for this population.

With the limitations in self-monitoring and reflective capacities among individuals with dementia, leveraging the environment becomes crucial, as it offers external sources of incentive motivation through observable behaviours, social norms, and contextual cues [23,24,25]. From a theoretical perspective grounded in Social Cognitive Theory, the social environment encompasses the dynamic and interactive settings (both physical and social) in which individuals operate and that shape their learning, behaviour, and development [15,22]. This environment includes immediate social relationships, cultural norms, institutional structures, and symbolic systems that regulate behaviour through observation, reinforcement, and cognitive mediation [16]. In the nursing home setting for people with dementia, the social environment is shaped by specific institutional norms, care regulations, and organisational routines that create a highly structured framework influencing residents’ behaviours and daily lived experiences [1]. These regulated norms and policies guide acceptable behaviours and impact motivation and feelings of efficacy within this unique social context. Importantly, cognitive mediation (i.e., the way residents interpret and assign meaning to social cues from staff, peers, and the environment) continues to play a vital role in shaping behavioural (and to a certain extent learning) responses, highlighting the intertwined psychological, physical, and social dimensions of the nursing home environment [16].

### 2.2. Response Facilitation as a Specific Form of Incentive Motivation Relevant for People with Dementia

Response facilitation is a specific form of vicarious incentive motivation that occurs when an individual observes another person engaging in an activity, leading to an increased likelihood of performing that behaviour themselves [19] or, in other words, by making potentially available behaviours more accessible [26]. It is a mechanism that refers to a modelled action by others that can serve as a (social) motivator for observers to also perform that same action [15]. Response facilitation could be a promising mechanism to influence and enhance engagement in desired behaviours among people with dementia. Leveraging the social environment, it offers a practical way to motivate individuals who experience cognitive impairments that limit traditional self-regulatory strategies. Response facilitation differs from other forms of social learning as it operates through distinct cognitive and environmental mechanisms and typically unfolds on a short timescale. In response facilitation, observing a model’s behaviour immediately or near-immediately increases the likelihood that the observer will perform a behaviour that is already part of their repertoire by temporarily raising its activation or salience rather than creating a new behavioural pattern and primarily influencing motivational readiness to act. In contrast, observational learning involves a multi-step process (attention, retention, reproduction, and motivation) in which the observed action is encoded and gradually consolidated into a new behaviour that can be performed later, often after repeated exposure and practice, rather than necessarily during the initial observation itself [15,20]. While research on errorless learning shows that learning new behaviour is possible for people with dementia [27], this process of observational learning can be more cognitively demanding, as it relies on capacities such as sustained attention, working memory, and error monitoring, which are frequently compromised in people with dementia. Therefore, response facilitation can be seen as a mechanism affecting motivation rather than learning. Furthermore, response facilitation differs from inhibition and disinhibition effects (also types of vicarious processes), which focus more on the observed effects of a certain behaviour on the modeller, in particular involving modelled actions that have moral, legal, or emotional implications. In contrast, response facilitation typically involves socially acceptable behaviour that fits in the (physical and social) context in which it is performed in [15,19]. Lastly, response facilitation differs from both observational learning and (dis)inhibition effects in that it can also be an automatic and unconscious form of mimicry as opposed to being a more social learning processes that require conscious attention and (extensive) cognitive processing [28]. This automatic process can be strengthened or less inhibited in people with dementia, as this process is usually regulated by goal-setting abilities and self-regulatory processes, which are often affected in dementia in general [29].

In the context of the nursing home in the Netherlands, where many people with moderate/advanced dementia live in groups with a shared living (and dining) room, it is particularly interesting to dive into the role that response facilitation can play in stimulating behaviour. Research has shown that modelling in 1-on-1 settings for people with dementia can aid in the (independent) execution of behaviour [30,31,32,33,34,35]. It consists of showing the desired behavioural steps, either by staff, by a volunteer (e.g., other resident), or by filmed behaviour of others [36,37,38,39]. This mechanism of response facilitation has also been shown to work for individual modelling without the use of verbal communication [40,41]. Furthermore, research on older adults without dementia and eating behaviour shows that the physical presence of others is not always required: eating in front of a mirror or in front of one’s own photograph increases both one’s appetite as well as enjoyment of food [42].

Interviewed experts [18] indicate that individuals in an advanced stage of dementia and/or those with vascular dementia (VD) and frontotemporal dementia (FTD) often require more environmental support to initiate or sustain social interactions. Key symptoms such as apathy, attention deficits, and impaired communication, which are prevalent in VD and FTD, significantly hinder social engagement. In contrast, Alzheimer’s disease (AD) is characterised by memory loss, wandering, and social withdrawal, which progressively worsen with the advancement of the condition [43,44,45]. Physical limitations like mobility issues and sensory impairments further exacerbate these challenges. For instance, mobility problems may reduce wandering but limit access to social spaces, while sensory impairments can intensify hallucinations or diminish responsiveness to environmental cues, leading to apathy or withdrawal [46,47]. Consequently, it could be that response facilitation (observing others engage in activities) may be less effective for individuals in advanced dementia stages or with VD/FTD due to pronounced apathy and reduced attention spans [18].

With the decline of intrinsic motivational processes due to dementia, one of the questions that arises is what kind of motivational techniques are successful in stimulating older adults with dementia in nursing homes to initiate and sustain certain types of behaviour. Specifically, the role of individual (e.g., staff interaction) or group processes (what is the effect of group behaviour on individuals) is relevant in this question. While research focuses a lot on individual guidance and personalization for people with dementia, the reality is that in practice, the shortage of qualified staff and volunteers often does not allow for repeated or extensive one-on-one settings during the day [48]. When somebody is observing somebody else or others experiencing pleasant effects of an activity, this can motivate one to also join in. This response facilitation can be a conscious or unconscious reaction to the social environment and can be seen as a form of ‘modelling’ behaviour by others [19,29].

The aim of this article is to explore the social contexts in which response facilitation, also called vicarious incentive motivation, can effectively engage older adults with moderate to advanced dementia in recreational activities in nursing homes.

The central research question guiding this study is: Within which social contexts can response facilitation effectively engage older adults with moderate to advanced dementia in recreational activities in nursing homes?

Based on previous research [1,18] we expect that response facilitation is well suited to the capabilities and situation of the older adult with moderate to advanced dementia living in nursing homes. We expect response facilitation to work in this population because it relies more on external environmental and social cues rather than internal self-regulatory processes, which are often compromised in moderate to advanced dementia.

Sub-questions include:

1. How do recreational activities naturally occur in psychogeriatric nursing homes?
2. Do non-participating residents display observable behavioural and/or physiological signs of engagement when observing individual activities, dyadic activities, or group activities?
3. How do observational and physiological indicators of engagement differ or align across the three social contexts?

## 3. Materials and Methods

This study employs a qualitative, scenario-based design to investigate response facilitation as a behaviour change mechanism for people with dementia in nursing homes. Two key concepts underpin the design: narratives and scenarios. Narratives are descriptive accounts of daily activities and experiences derived from detailed fly-on-the-wall observations and unstructured interviews with residents and staff [49,50]. These narratives capture the lived experiences and contextual interactions of individuals with dementia, highlighting typical behaviour patterns and environmental influences. Specifically, the narratives focus on the social environment and interaction in relation to performed activities for people with dementia.

The narratives are of particular importance due to the specific (social, organisational, and physical) circumstances of the nursing home for people with dementia that influence the lived experience of the residents profoundly. By constructing narratives, a scenario for testing response facilitation in different social contexts can be constructed that closely resembles the daily activities of the residents. This allows us to look at how the mechanism can work in different social contexts, rather than focusing on the effect that ‘novel’ activities, people, or social constellations may have on behaviour in this target group. Based on these narratives, response facilitation is translated into a scenario with three types of ‘social’ presence: (1) activity performed by another resident only; (2) activity performed by another resident and one care professional or volunteer; and (3) activity with a group of residents and one care professional leading it. The reactions (physical and physiological) of other residents are observed and measured during these scenarios. The scenarios thus represent simplified, controlled versions of daily activities that allow specific aspects of the response facilitation mechanism to be explored.

The relationship between narratives and the scenario is foundational: narratives informed the content and structure of the scenario by providing realistic, context-sensitive frameworks. This ensures the scenario closely mirrors actual daily routines and events, preventing testing the ‘novelty’ of the activity or setting rather than the response facilitation mechanism.

In this study (Table 1), (1) fly-on-the-wall observations and informal interviews are used to construct (2) narratives. These narratives then guided the creation of (3) a scenario involving structured and sequential facilitation steps. The scenario was tested in naturalistic settings, measuring physical and physiological engagement through both observational and physiological data, providing insights into whether and how response facilitation strategies affect doing recreational activities in people with dementia in nursing homes.

### 3.1. Case Studies

In the Netherlands, long-term care policy is strongly oriented towards enabling older adults, including people with dementia, to live at home for as long as possible with community and home-care support. Admission to a nursing home, therefore, generally occurs only when living at home is no longer safe or feasible due to risks to the person or others, or because the intensity of care required exceeds what can be provided in the home setting. As a result, residents in Dutch nursing homes typically have moderate to severe cognitive impairment and high care needs.

Nursing homes commonly distinguish between somatic wards, which primarily accommodate residents with predominantly physical and somatic conditions, and psychogeriatric wards, which provide 24 h supervision and care for residents with dementia and associated behavioural and psychological symptoms. These Dutch psychogeriatric nursing homes are typically organised around small-scale living units with shared living and dining rooms, where daily life is structured by communal routines, while residents maintain private bedrooms [51]. This study was conducted in collaboration with three care organisations, involving four psychogeriatric nursing home locations, of which in each one ward (living room with hallway and adjacent resident rooms) was selected as a case study. Note that these ‘wards’ in Dutch nursing homes represent residential living environments with integrated care, rather than hospital-like medical units. The selection of these wards was based on practical factors, including staff availability and ethical approval for resident participation.

### 3.2. Participants

Dementia diagnosis, dementia subtype, and severity were determined by the nursing home geriatricians as part of routine clinical assessment and documentation (e.g., based on medical history, neuropsychological evaluation, and multidisciplinary team input). Only residents with a documented diagnosis of dementia and judged by the geriatrician as having moderate to advanced dementia were eligible (which excluded mostly inhabitants that had both dementia and another illness or conditions that required intensive care, as in principle, all residents had dementia). Permission from informal caregivers was obtained to disclose diagnostic information (dementia type and severity category) to the research team for research purposes.

The inclusion criteria were:

- residence on one of the participating psychogeriatric wards;
- a documented diagnosis of dementia with moderate to advanced severity according to the nursing home geriatrician;
- ability to move independently through the communal living area (with or without mobility aids such as a walker or self-propelled wheelchair), so that potential engagement with ongoing activities in the shared spaces could be observed;
- informal caregiver consent for participation and data collection (observations and physiological measurements);
- absence of clear and persistent objection by the resident during day-to-day interactions with researchers (ongoing assent).

Exclusion criteria were:

- being bedbound or fully dependent on staff for transfers and mobility, such that the resident could not independently move through or remain in the communal living spaces;
- severe sensory or motor impairments that would preclude meaningful observation of responses to activities in shared spaces;
- acute medical instability or superimposed delirium, as judged by the clinical team;
- refusal of informal caregivers or clear/repeated refusal by the resident to be observed and/or to wear sensors.

The sample reflected the prevalence of Alzheimer’s disease (AD), which accounts for 60–70% of dementia cases, alongside vascular dementia (VD), frontotemporal dementia (FTD), Lewy Body Dementia (LBD), Parkinson’s Disease Dementia (PDD), and mixed types involving AD and VD [52]. Participant details regarding health status, hobbies, visitors, and previous occupations were recorded to contextualise observational and physiological data. For confidentiality purposes, pseudonyms were assigned to participants, and nursing homes were referred to as case study 1, 2, 3, or 4.

### 3.3. Methods Step 1: Mapping Daily Activities

Fly-on-the-wall observations were utilised to map daily routines within the nursing homes and to examine resident behaviours during structured scenarios. This method was chosen to address limitations of interviews and surveys due to memory and orientation issues of people with dementia and potential reflection biases of their caregivers [53,54]. Observations focused on six key elements: participants, actions, relationships, contexts, locations, and mood/agitation levels [55]. Data were recorded on annotated floorplans linked to observation lists and later digitised for analysis (Supplementary File S1).

The observation list was refined based on prior research [1,56] and included detailed tracking of mood changes, agitation levels, and interactions between residents and staff. A total of 4137 unique observations were made across the four case studies (*n* = 21), highlighting how structured care routines and environmental design influence social interactions and leisure activities among residents with dementia. These observations formed the bases for the narratives that showcase typical behaviours in various social contexts. To ensure reliability of observational assessments, two researchers independently completed the observation scales in real time, then cross-checked their ratings immediately after. This was done for 30 min of each day of observations. Subsequently, care professionals on duty reviewed a randomly selected subset of two measurements per participant to validate the accuracy of researcher interpretations. No substantive disagreements arose between care staff and researchers regarding the observed emotional or behavioural states.

The observational scale scores were then integrated with the broader fly-on-the-wall observation data of behaviour, creating a comprehensive dataset that captured both the activities performed and the associated emotional, mood, and agitation responses. Analysis proceeded through two complementary approaches: thematic analysis to identify recurring patterns in resident behaviours and emotional responses across scenarios and contextual analysis to examine the situational, social, and environmental conditions that characterised these responses.

The researchers conducting the observations had prior experience with similar observational and physiological measurement methodologies in studies with people with dementia, e.g., [1,56,57]. To ensure rigorous and transparent interpretation, the thematic and contextual analysis of this data used for the narratives was conducted collaboratively: the first author performed the initial coding and thematic identification, which was then systematically reviewed, discussed, and refined through iterative feedback sessions with the other co-authors. This collaborative approach allowed multiple perspectives to be brought to bear on the data, reducing the risk of individual interpretive bias. Throughout the analysis, the research team maintained explicit awareness of their own assumptions and interpretive choices.

### 3.4. Methods Step 2: Narratives for Constructing the Scenario

Narratives were developed from these detailed fly-on-the-wall observations to capture the daily routines, behaviours, and social interactions of nursing home residents with dementia. These narratives served as a foundation for designing scenarios that tested response facilitation. Observational methods were chosen over interviews due to the cognitive and linguistic challenges often faced by individuals with dementia, which can limit their ability to accurately recall or articulate experiences [58,59]. The use of narratives provided a nuanced understanding of how residents engaged with their (social) environment and others in various contexts. The two observing researchers discussed their observations and field notes collaboratively throughout data collection to identify convergent patterns and ensure consistency in how activities and social contexts were characterised. Narrative construction subsequently involved iterative review and refinement by the whole research team to ground the accounts in collectively validated observational data.

The narratives played a crucial role in scenario development for two primary reasons. First, they ensured that scenarios were closely aligned with familiar daily routines, reducing the risk of testing novelty effects rather than the response facilitation mechanism. Second, they provided a detailed account of the social, organisational, and physical settings within the nursing homes, enabling the translation of theoretical concepts into practical and contextually relevant scenarios [49]. This approach allowed for realistic experimentation while maintaining ecological validity.

The narratives are selected based on two selection criteria: the described narrative depicts (1) the engagement of one or more residents in a leisure activity and (2) (a variety in) the social context, e.g., alone, with a caregiver, with family/visitors, within a group. The narrative describes independent and assisted activities and highlights the role of social (e.g., staff support) environments [16].

### 3.5. Methods Step 3: Scenarios

From these narratives, three scenarios are formulated. The narratives are both used to further define the precise translation of response facilitation to real-life experiments as well as to embed this scenario into the physical and socio-organisational context of the nursing homes. This led to three scenarios that are embedded in the daily life of people with dementia.

The scenario design incorporated three types of social contexts to explore response facilitation strategies in real-life settings. Each of these scenarios includes one or more residents doing the ‘initial’ leisure activity and one or more ‘observing residents’ who are the actual participants in the study. Their reaction is what is of interest for this research. The social contexts examined are:

1. Individual Activity: One resident engaging in an activity independently, observed by others (i.e., one or more participants in the study).
2. Dyadic Activity: A resident performing an activity alongside/with a caregiver or volunteer, offering additional social cues for engagement, again observed by others (participants).
3. Group Activity: A structured group setting where multiple residents participated in an activity led by a caregiver or volunteer facilitator, observed by others (participants of the study).

Facilitation consistency was maintained through the physical and organisational context across wards (communal living areas, daily routines), while specific activities and staff behaviours varied naturally to reflect ecological validity. The scenario design aimed to isolate social context effects (individual vs. dyadic vs. group) from facilitator or activity content effects, prioritising observer responses over uniform intervention delivery. Standardising activities across scenarios would have prevented addressing the main research question, because if these activities were ‘new’ or not habitually done by the staff, the question is whether novelty or social context was tested.

Each scenario lasted between 10 and 120 min, following the organisation’s daily routines and natural activity schedules. Individual and dyadic activities typically lasted shorter periods (10–40 min), while group activities often extended longer (40–120 min), reflecting how these activities naturally unfolded in the nursing home. Activities were selected in consultation with nursing home managers and care staff based on routines already established within each ward (before the start of the study), supplemented by insights from the observational data and staff interviews (from the fly-on-the-wall observations and informal interviews with staff); this approach ensured scenarios reflected genuine, familiar practices rather than novel or experimental interventions.

Staff facilitation during scenarios (e.g., verbal encouragement or invitations to join the activity) was left to care staff, who used their typical communication approaches with residents. The specific content and tone of staff prompts were recorded in video and detailed observational notes, allowing for later analysis. While the specific activities and staff interactions varied across wards and scenarios (reflecting the natural diversity of each nursing home’s environment and resident population), the three social context types (individual, dyadic, and group) remained structurally identical across all case studies, and the physical spaces (communal living areas) were similar in function and furnishing, though not identical in design or layout. Volunteers and care professionals were instructed not to do anything different from what they would normally do, only asked if they could do a leisure activity with one or more of the residents. In the case of the individual activity scenario, no additional prompting or facilitation was used; instead, researchers observed residents who naturally engaged in a leisure activity independently and recorded how other residents present in the communal space responded to this ongoing activity.

Observational data on mood and agitation were collected using validated tools such as the Observed Emotion Rating Scale (OERS) and the MEDLO scale. These scales assessed emotional states (e.g., pleasure, anger, anxiety) and agitation levels (e.g., verbal outbursts, physical restlessness) through a 5-point Likert scale. These observations are done over time, recording all changes in mood, agitation, or behaviour—leading to detailed outcomes over time (rather than over fixed timed intervals). Two researchers independently recorded these observations in real-time, cross-checking their assessments post-scenario to ensure reliability. Additionally, care professionals on duty reviewed two randomly selected measurements per resident to validate accuracy.

**Figure 1:**
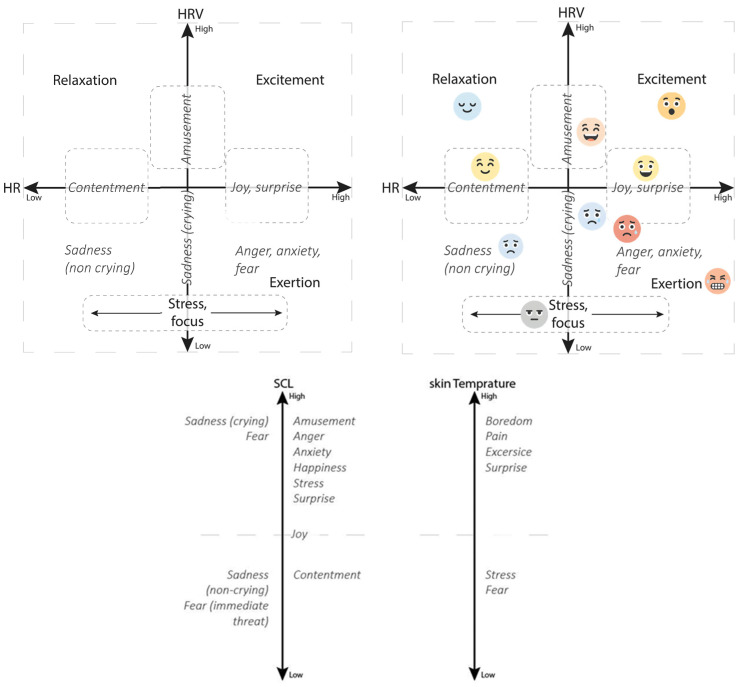
This figure, partly taken from [56], shows the interpretation of the physiological data. Where it says HR and HRV, respectively, PR and PRV can also be read. The graph with the emoticons shows the visualisation of how HRV/HR (PRV/PR) data can be interpreted into different moods. The symbols are used to indicate changes in physiological data in the graphs in the results section. When there are multiple interpretations possible of the same values (e.g., low HRV and high HR), observational data of mood and behaviour further determine the interpretation.

Physiological data were collected using wearable sensors to measure heart rate (HR), heart rate variability (HRV), pulse rate (PR), pulse rate variability (PRV), skin conductance level (SCL), and temperature. These physiological indicators provided objective insights into stress levels and emotional responses during the scenarios (see Figure 1). Baseline physiological data were recorded during rest periods for comparison with scenario-specific measurements, allowing for individualised interpretation of stress or arousal levels. Interpretation of the physiological data is done based on earlier research with these wearables and the target group [1,56]. Important to note in the interpretation of the graphs used in this article is that all presented ‘states’, as seen in Figure 1 (e.g., amusement and exertion), can be interpreted as relative to previous states. While also shown in relation to a baseline, the movements as plotted in the graphs in the results section present a ‘movement towards’ a state, rather than final and definitive end-states.

To ensure precision in data analysis, all observational and physiological data were synchronised with video recordings of the scenarios. This allowed researchers to match physiological changes with specific moments during activities, providing a comprehensive understanding of how response facilitation influenced behaviour. Baseline data collection was conducted over two days to minimise participant fatigue, with scenario testing scheduled separately from other organised activities. During each scenario, two researchers positioned themselves at different angles within the communal space, maintaining sufficient distance to minimise interference while capturing detailed observational data. All scenarios were video-recorded to enable synchronisation of behavioural observations with physiological data and to allow post hoc review and validation of researcher interpretations. Immediately following each scenario, the two observing researchers cross-checked their observational assessments to ensure consistency and to note any divergences in interpretation. Any differences were either discussed with the staff on duty or cross-checked with the video images when those were processed later.

### 3.6. Ethical Considerations

For this study, the Ethical Review Board of the Eindhoven University of Technology has approved the study beforehand. Informed consent was obtained from the informal caregivers of the nursing home residents participating, which was mediated by the care organisations. During the scenario and during the application of the sensors, consent about wearing the sensor was asked from the resident. That is to say, if the participant did not want to wear it, it was not put on. 

## 4. Results

The Results section presents the empirical findings of this study, examining how response facilitation operates within varied social contexts among people with dementia in nursing homes.

Of the 44 potential residents on the selected wards, 11 were excluded based upon meeting the first three exclusion criteria or failing to meet the first three inclusion criteria. Another 12 were excluded because the family did not give informed consent. This led to 21 residents being included. An overview of participants, type of dementia and severity can be found in the results section, Table 2. To better understand the daily activities of nursing home residents with dementia, and uncover patterns in their behaviour, 4137 unique observations were conducted across communal areas in four psychogeriatric wards (*n* = 21) that formed the basis for the narratives below.

Physiological measurements (HR/PR, HRV/PRV, SCL, and temperature) were not consistently available for all participants due to technical issues or participant refusal, resulting in a sample size of *n* = 15 who have completed at least one out of the three possible scenarios. Firstly, some participants expressed discomfort or refused to wear the sensor during specific scenarios (at other times, they did wear them; no resident consistently refused to wear the sensor when asked). Secondly, technical issues arose with the sensors themselves. For example, sensors occasionally failed to capture sufficient data due to improper placement (or shifting) under clothing or inconsistent skin conductivity, which can vary with age and health conditions [56]. Additionally, certain participants removed the sensors during the experiment, either unintentionally or deliberately. This behaviour was observed in cases where residents seemed to become restless or distracted during activities, leading them to adjust their clothing or accessories. As it was not always clear what the reason was, the data were not collected (e.g., no way to distinguish if the sensor shifted or the conductivity of the skin was the reason), which is indicated with a * in Table 2 for all reasons. Observational data on mood and agitation were collected for all participants during all observed scenarios using validated scales (Supplementary Files S1 and S2).

### 4.1. Fly-on-the-Wall Observations and Narratives

The goal of the observations and narratives was to answer sub-question 1: ‘How do recreational activities naturally occur in psychogeriatric nursing homes?’. The observations were therefore analysed with a focus on several key aspects: (a) the type of activity performed, (b) whether it was done individually, with somebody else, or within a group, (c) who initiated the activity, and (d) mood and agitation levels observed during engagement. The majority of nursing home residents with dementia typically have Alzheimer’s disease (60–70%) or vascular dementia (15–20%), which was reflected in the study sample (see Table 2). The narratives tried to include residents with a varying range of severity levels and symptoms, aiming to illustrate diverse experiences of social interaction. These narratives are not exhaustive but serve to exemplify how leisure activities are embedded within social contexts.

Five out of thirty-three narratives relating to hobby/recreation activities were selected as illustrative examples of how response facilitation operates in different social contexts (individual, dyadic, group). The two observing researchers initially identified narratives that authentically represented the typical pace, rhythm, and social dynamics of daily life in the nursing homes, based on their field observations and informal interviews with caregivers. These preliminary selections were then reviewed by the research team to ensure that the five narratives collectively captured the diversity of social contexts (individual, dyadic, group) and reflected genuine, recurring patterns of activity initiation and engagement rather than exceptional or atypical occurrences. These narratives highlight variations in how activities are initiated or sustained and by whom, as well as the social context in which they took place. Section 4.3 further outlines these contexts, showcasing how residents respond to scenarios involving individual actions, dyadic interactions with caregivers or volunteers, and group settings. Narrative 1: Douglas (Douglas, and all other residents’ or staff’s names are fictional for privacy purposes) reads the paper (resident D, case study 1, living room, moderate-severe dementia, AD):

It’s a quiet afternoon in the living room after lunch. Douglas sits at the table, flipping through the morning newspaper, although not reacting physically or verbally to what he ‘reads’. Edward sits next to him and occasionally glances over to Douglas, sometimes craning his neck to glance at the paper too. After a few minutes, Edward asks, ‘Can I see the paper?’ Douglas pauses briefly before folding it and sliding it across the table. Douglas now sits at his spot, not doing anything, just glancing around the room. Edward spends the next 15 min bent over the paper in silence. Whether he’s reading or dozing off is unclear, he is completely bent over the newspaper, his nose almost touching the paper. Eventually, he straightens and he neatly folds it, places it back on the table, and pushes himself slightly from the table with his wheelchair.

Narrative 2: Annie knitting (resident A, case study 3, living room, moderate-severe dementia, AD + VD, and resident B, case study 3, living room, severe, AD:

It’s a quiet mid-morning in the living room of the nursing home. Annie strolls in with her walker and in the basket in the front, she brings some knitting supplies. She sits down by the window, knitting quietly as sunlight streams in. A couple of staff members pass by, greeting Annie warmly: ‘Good morning.’ She looks up briefly, smiles, and returns to her knitting. Three other residents acknowledge her with nods or brief hellos but don’t comment on her work. Annie’s focus is interrupted when Ben enters. He walks straight to her, bypassing the dining table without a word. ‘You want to go for a walk?’ he says. Annie hesitates, holding up her knitting as if to explain that she’s busy. Ben waits silently, showing no reaction to her reluctance, but just says again: ‘I think we should go for a walk?’. After a moment, Annie begins carefully packing up her supplies. Once ready, Annie joins Ben, with the knitting again in the basket of her walker: ‘I have to put this in my room first! Let’s just go that way now first’.

Narrative 3: Edward and a volunteer take a ‘walk’ (resident E, case study 2, living room/hallway, mixed type dementia, moderate):

Edward is sitting quietly in the living room, as he often does. A volunteer enters and begins chatting with another resident nearby before turning her attention to Edward. ‘[comment on enjoying sitting in the living room] You do need to move more, Edward’ she says with a friendly smile. Edward nods in agreement: ‘Perhaps’. No one else in the room reacts to their interaction as Edward adjusts himself in his wheelchair and follows her out. In the hallway, the volunteer remains focused on Edward, engaging him in light conversation as they pass others. They exchange brief greetings with residents and staff along the way, but remain in conversation during the activity. When they are standing at the window looking outside, a resident from another group joins them silently. When Edward and the volunteer leave that spot, the other resident stays looking intently out the window.

Narrative 4: Byron reads the paper with a care professional (resident B, case study 2, living room, mixed type dementia, moderate):

Byron sits in the living room with today’s newspaper lying in front of him. Earlier, he flipped through it, but now he’s simply staring around the room. A care professional approaches him and asks, ‘What’s in the paper today?’ Byron shrugs and replies, ‘I don’t know.’ The professional sits beside him, opens the paper to the front page, and they begin reading together. When they reach a story about the royals, Byron shakes his head. ‘Not my thing,’ he says plainly. ‘I’d rather read other stories.’ As they continue, two residents pause behind them, curiously glancing over their shoulders at what they’re doing but not engaging directly. The care professional doesn’t notice as she remains focused on Byron, chatting amicably about what’s in the paper while others move around the room.

Narrative 5: Bernice joins bingo (resident B, case study 1, activity room, moderate-severe, AD):

The living area is calm after breakfast, with residents scattered in quiet activity. Bernice sits at the central dining room table with her coffee when two staff members enter and announce: ‘Time for bingo!’ They approach specific residents, including Bernice, inviting them personally to join. Not all people are asked, just the ones who are on the schedule. Bernice smiles and agrees, rising to join two others who were also invited. The remaining residents watch this unfold without comment. Bernice and the small group are led to a separate activity room arranged specifically for bingo. The atmosphere is lively and structured, with care professionals warmly welcoming participants and guiding them to their seats. As the game begins, care professionals help residents find numbers on their cards and share small jokes about their luck. Not all residents seem to grasp the goal of the activity (they do not stamp any of the called numbers), but they are smiling and looking at other residents, clapping when someone has a bingo. Bernice also claps enthusiastically, smiling despite not winning herself.

### 4.2. Constructing Scenarios

During these narratives, key elements for developing the scenarios were the (social) context in which the activity took place (by themselves, with somebody else, or in a group) and with whom (with other residents, with caregivers, with family/visitors), the physical context, the type of activity, time, and (if the resident took the) initiative. Table 3 shows an overview of these two key elements per narrative that have been used to develop the scenario.

To answer sub-question 1 (how do recreational activities naturally occur in psychogeriatric nursing homes?), observations revealed that most recreational activities involved either one-on-one (resident with caregiver or volunteer) or group settings, with only a few self-initiated individual activities. Caregivers included nurses, physiotherapists, and well-being specialists; to reflect authentic practice, developed scenarios primarily featured care professionals, with volunteers included only when they regularly facilitated leisure activities across multiple residents (gathered from observations and informal interviews).

Interactions with activities were more frequent in central communal areas (e.g., the dining table in the living room) compared to hallways or separate spaces. For example, Annie’s knitting by the window generated minimal interaction from others, while Douglas and Edward’s newspaper exchange at the central table prompted some responses (Edward’s glances and request). Byron’s reading with a care professional attracted curious glances only when residents walked directly past the table. Scenarios were therefore conducted in the living room around the central dining table.

Timing also mattered: mid-morning, early afternoon, and early evening saw higher resident presence in shared spaces, whereas early morning and late evening coincided with residents retreating to rooms or attending external activities. Scenarios were scheduled during these high-occupancy periods. By situating activities in central areas during ‘busy’ times with familiar facilitators (caregivers/volunteers), scenarios aimed to closely mirror daily life while enabling observation of how non-participating residents responded to others’ engagement.

### 4.3. Observational Outcomes of Scenarios for Response Facilitation

The results from the observations of behaviour, mood and agitation (see Supplementary Files S1 and S2) are reported on below and aimed at answering the first half of the second subquestion: ‘do non-participating residents display observable behavioural and/or physiological signs of engagement when observing individual activities, dyadic activities, or group activities?’. At least one individual, one dyadic, and one group activity were observed in all case studies, but the group activity for case study 1 was not visited by any of the participants of this study (only other residents), so it was left out of the results below.

Individual: Across all four case studies, non-participating residents exhibited minimal to no active engagement with individuals engaged in solitary activities, though subtle contextual differences emerged. In Caroline’s scenario (Case Study 1), her laundry folding and singing at the central table drew brief glances from Edward, who eventually joined her briefly in folding the laundry, while Bernice continued conversing with others without acknowledging her. Byron (Case Study 2), reading alone at the dining table, attracted sporadic glances from Daisy and eventually him asking for the paper (‘Can I?’), but most residents (e.g., Ada and Charlotte) remained disengaged, focused on meals or staring into space. Edith (Case Study 3), reading a newspaper at the table, prompted conversation about the newspaper between Dora and another care professional, while Annie did not react and stared passively at another spot in the room. Delilah (Case Study 4), reading magazines centrally at the dining room table, elicited no observable reactions from others; Audrey repeatedly entered/left the space without interaction, and Blanche peeled potatoes nearby without glancing at her activity.

Dyadic: In Douglas’s (Case Study 1) game with a welfare professional, one resident is already sitting at the table (Edward), and two other residents (Bernice and Caroline) sit down after the game starts. Edward starts off angrily declining to join in the beginning of the game, after which he looks around the room, but not at the game. Halfway through, when Bernice and Caroline are already verbally involved with Douglas and the welfare professional, he starts intently looking at the game and the interaction. Bernice enters the room two minutes after the game started and starts out by sitting down and looking intently at the game. First, she comments on the game a couple of times; when the dice falls down, she picks it up. She is not invited to join the actual game by Douglas or the welfare professional but keeps being verbally engaged. She pokes Caroline and tries to get her to ‘join’ too, but Caroline only looks at the activity and does not physically or verbally interact. Similarly, in Charlotte’s gardening activity (Case Study 2), where a volunteer helps Charlotte plant some bulbs in a pot, Byron watched intently for several minutes before resuming staring at something else and seemingly dozing off, while other residents remained disengaged (Ada, Daisy). During Joe’s (Case Study 2) puzzle with a care professional, other residents displayed limited engagement. Byron glanced at the puzzle pieces briefly but did not approach or comment, while others in the room (including Daisy) remained focused on their own activities. When a volunteer is reading the paper with Jane (Case Study 2), Byron, Charlotte, and Elise are in the living room. Ada and Daisy walk past in the hallway and look through the glass windows into the living room. Daisy (from the hallway) and Byron (from the opposite side of the table) seem to glance at the reading activity. Both avert their eyes, and neither them nor the other residents further react to the activity. In John’s (Case Study 3) Christmas card-making scenario, nearby residents showed some involvement. Dora and Edith verbally commented on John’s card (‘ well done!’ and clapping), and Edith also looked at the materials displayed. Other residents, Annie and Clara, sit at the table, and Ben and Frank walk past multiple times, but none of them react in any way. In Eleanor’s (Case Study 4) phone call with her daughter (guided by a care professional), nearby residents demonstrated more active responses. Audrey reacts to the interaction between Eleanor and the care professional and them looking at the phone (‘What are you looking for?’) but doesn’t look or talk to them during or after the call anymore. The other residents (Delilah and Constance) do not react to the conversation.

Group: In group activities, non-participating residents displayed varying degrees of engagement, ranging from passive observation to active involvement. In the Table Ball game (Case Study 2), a volunteer initiates a game of table football for a group of residents, including Elise. At first, four residents are actively involved, but the other two—including Elise—do not seem to respond physically or verbally. This changes once a ball is rolled to her by another resident. Elise is not in time to catch the ball, and it rolls off the table, but she laughs, and when the ball is picked up by a care professional and handed back, she rolls it to another resident. From that point on, she is grinning and using her facial expressions (‘shock’, enthusiasm, concentration) at the game and interaction at the table. During the proverb guessing game (Case Study 3), residents seated at the table actively participated, while others in the living room occasionally glanced toward the activity or moved closer to observe. For example, Ben wandered through the room, intermittently looking at the table but not joining. And Frank initially engaged in unrelated tasks (e.g., arranging cushions) and walking around before unexpectedly participating by answering a question loudly. In the ball game (Case Study 3), residents who initially declined to join, such as Annie and Ben, frequently glanced at the game (but kept verbally declining to join) and eventually did participate when a staff member rolled a ball towards them. The Christmas activity (Case Study 4) elicited mixed responses from non-participating residents. While some residents at the table engaged in light conversation about the materials, others displayed disinterest or left the room entirely. For instance, Eleanor commented dismissively on the activity before leaving, while Constance briefly interacted with participants but became upset and withdrew.

### 4.4. Combining Physiological and Observational Data of Scenarios for Response Facilitation

This section presents results to the second sub-question: ‘do non-participating residents display observable behavioural and/or physiological signs of engagement when observing individual activities, dyadic activities, or group activities?. The section presents four case examples that demonstrate the integration of physiological measurements with observational data to reveal nuanced insights into physical responses and cognitive engagement during individual, dyadic, and group activities. At the end of the section, a complete overview of behavioural and physiological data per resident can be seen (Table 4). The selection of these cases was purposefully chosen to address four elements arising from the general data set: (1) whether identical observed behaviours can produce divergent physiological responses; (2) how physical proximity and observational positioning relate to engagement; (3) whether physiological indicators of cognitive engagement precede or follow verbal participation; and (4) how apparent incongruence between verbal expressions and physiological states can illuminate authentic emotional responses.

**Figure 2:**
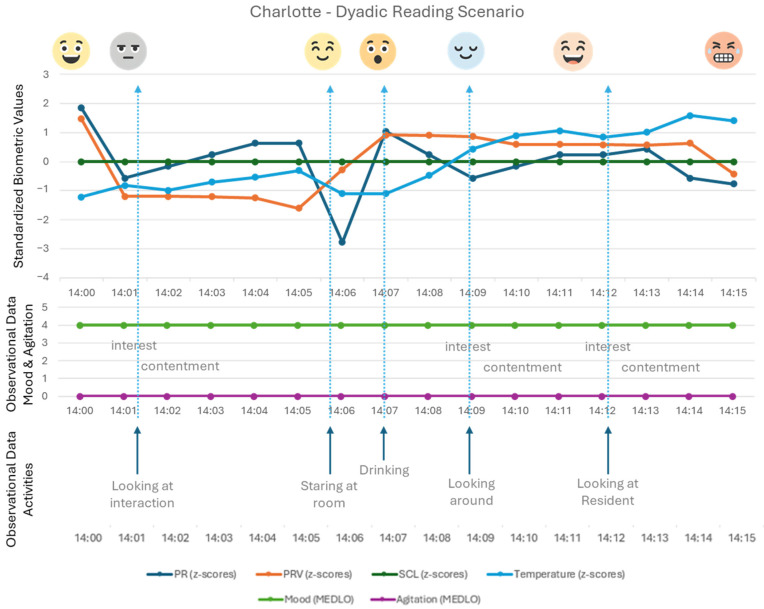
Graph view of physiological and observational data (mood, behaviour) of a scenario in which Charlotte (Case Study 2) ‘responds’ to a dyadic scenario (another resident reading a newspaper with a caregiver), visualised over time. All physiological values are standardised measurements (z-scores).

Figure 2 shows an example where the same observed behaviour (‘looking at’) is seen to result in different physiological data (interpretation, see Figure 1). During the observations, Charlotte is seen looking at different elements in the room at least four times (see Figure 2). The interpretation of the physiological data above the graphs shows how Charlotte is focused (mentally engaged), observing social interaction (between the caregiver and other residents). When she is staring at another resident (twice) who is sitting at the table or is looking around in the room, one can see contentment, amusement, and relaxation.

**Figure 3:**
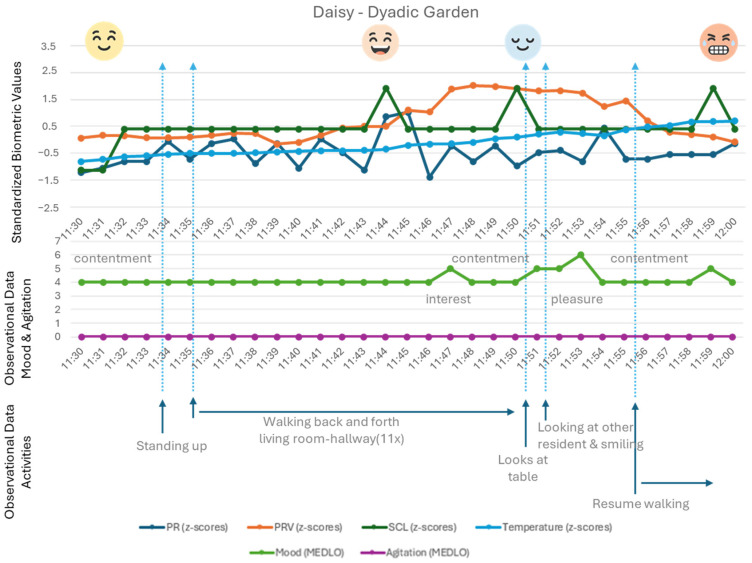
Graph view of physiological and observational data (mood, behaviour) of a scenario in which Daisy (Case Study 2) ‘responds’ to a dyadic scenario (another resident doing indoor gardening with a volunteer), visualised over time. All physiological values are standardised measurements (z-scores).

Figure 3 shows the physiological data of Daisy, who is a resident who walks a lot through the hallways of the ward. First of all, one can see Daisy walks a lot, as this observed walking back and forth does not result in her physiological values showing exertion. In fact, her data shows that when she is looking through the windows at the interaction taking place, as well as when she walks through the room and looks at the interaction, she is relaxed and amused. It seems that interaction and these (dyadic) activities are noticed, perhaps even enjoyed, but in the case of Daisy, do not trigger any mental engagement.

**Figure 4:**
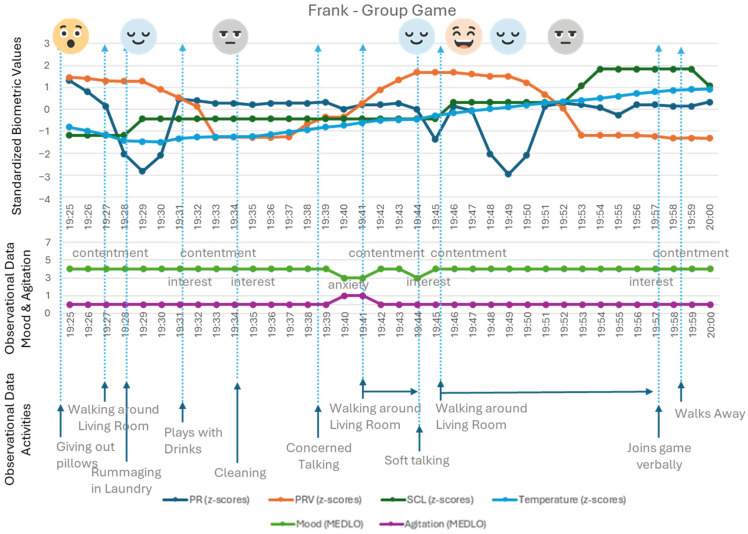
Graph view of physiological and observational data (mood, behaviour) of a scenario in which Frank (Case Study 3) ‘responds’ to a group scenario (a group of residents doing a puzzle game), visualised over time. All physiological values are standardised measurements (z-scores).

The data in Figure 4 shows the physiological data of Frank during the ‘guess the saying’ game played by a group in the living room. Frank rarely talks and wanders a lot. During the game, he is not sitting down but wandering around the group. He starts by ‘dusting off’ pillows and rearranging them, cleaning them with a cloth—his values showing excitement during this activity. Later on, he is strolling around the group, softly talking to himself, looking anxious, while his physiological values do not reflect this. Roughly five minutes before he verbally engages with the game (guessing the correct and very obscure answer), his values go from relaxation to cognitive engagement. His observations of mood and agitation do not show this. 

**Figure 5:**
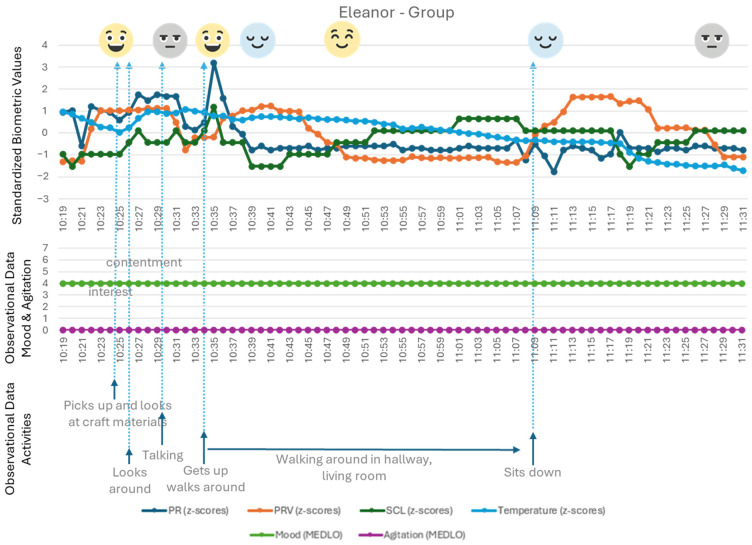
Graph view of physiological and observational data (mood, behaviour) of a scenario in which Eleanor (Case Study 4) ‘responds’ to a group scenario (a group of residents making Christmas cards), visualised over time. All physiological values are standardised measurements (z-scores).

The data in Figure 5 shows Eleanor’s physiological values during the group activity in the living room (making Christmas cards). Eleanor is invited by the care professional to join verbally (‘talking’), but she scoffs and indicates she is absolutely not interested in the activity; she thinks it’s ‘stupid’. That being said, before this remark, she picks up the materials and looks at them (values indicating amusement), and during the negatively loaded verbal exchange, her physiological values also show amusement, indicating that she is experiencing positive emotions during this exchange. Perhaps the verbal interaction is enjoyable to Eleanor, regardless of the content of the interaction.

To answer sub-question 3 (how do observational and physiological indicators of engagement differ or align across the three social contexts?), Table 4 presents observational and physiological data collected across all 39 scenarios (*n* = 21 residents with observational data across all 39 scenarios; *n* = 15 with physiological data in 21 scenarios). Of these scenarios, 7 were group activities, 11 individual, and 20 dyadic (18 scenarios had behavioural data only).

Individual scenarios (*n* = 11) elicited the fewest and weakest responses of all contexts. Behaviourally, 3 residents showed any type of engagement: one joined physically, another combined a physical response with verbal comments about the activity, and a third offered a single verbal comment. Physiologically, among the 11 scenarios, only 1 included a resident with a physiological reaction: a brief episode of concentration when looking at the activity.

Dyadic scenarios (*n* = 20) seemed to yield moderately stronger effects. Behaviourally, in 4 scenarios, residents responded with 3 verbal reactions (e.g., short comments on the activity and a question regarding the activity) and 1 combining verbal and physical actions (i.e., attempting to join the game). Physiologically, 8 reactions were recorded across residents (2 excitement, 5 focus/concentration, and 1 amusement). Congruence between behavioural and physiological data remained limited; only 2 of these 4 behavioural responders also showed physiological change, while the other 2 displayed no deviation from their baseline or previous physiological states.

Group scenarios (*n* = 7) relatively generated the most robust signals across both measures. Behaviourally, in 4 out of 7 scenarios, a resident engaged through 1 physical response, 1 physical as well as verbal combination, and 2 verbal contributions (e.g., joining the game, active participation in conversation). Physiologically, 5 reactions occurred (2 amusement, 2 excitement, 1 engagement), which aligned more with the behavioural observations than the dyadic scenarios. In the group context, the 5 physiological reactions included those where the behavioural response was also seen.

When combining these indicators, congruence varied across contexts, revealing differences in how observational and physiological measures aligned. In individual scenarios, both measures showed consistently low engagement with great alignment (3 behavioural responses and 1 physiological). Dyadic scenarios demonstrated limited overlap: of the 4 behavioural responses, only 2 also showed physiological change, while 2 behavioural responders displayed no deviation from baseline, and 6 additional physiological reactions occurred without behavioural signs. Group scenarios showed again stronger alignment: of the 5 physiological reactions, 4 corresponded to the behavioural responses observed, suggesting more consistent multi-modal engagement under these conditions.

To answer how observational and physiological indicators of engagement differ or align across the social contexts, observational and physiological indicators show patterns (group strongest, dyadic moderate, individual weakest) but differ substantially in detection. Individual contexts showed a high measure of congruence but low overall engagement (3 behavioural and 1 physiological). Dyadic contexts revealed the greatest divergence (12\ total responses across measures, only 2 overlapping). Group contexts demonstrated the strongest responses (4 behavioural and 5 physiological) and the highest congruence (physiological reactions matched behavioural ones). These patterns support the idea that single-modality assessment may miss engagement (particularly in the dyadic scenarios) and that group contexts provide conditions where both overt behavioural and physiological responses most consistently co-occur.

## 5. Discussion

### 5.1. Summary of Main Findings

This study examined how response facilitation, as a specific form of vicarious incentive motivation grounded in Social Cognitive Theory, may support engagement in recreational activities among nursing home residents with moderate to advanced dementia. Across four psychogeriatric wards, fly-on-the-wall observations and narratives showed that everyday recreational activities predominantly occurred either as one-to-one interactions between residents and caregivers or volunteers, or as organised group activities, with relatively few self-initiated individual activities. Scenario data confirmed that individual contexts elicited the weakest responses across both behavioural and physiological measures, dyadic contexts produced moderate effects with substantial measure divergence, and group contexts generated the strongest and most congruent multi-modal engagement. These findings demonstrate that response facilitation manifests most reliably in structured group settings (where behavioural and physiological indicators align most consistently), while single-modality behavioural assessment seems to underestimate the prevalence and complexity of internal engagement captured through physiological measures.

### 5.2. Narratives: Recreational Activities and Contexts in Everyday Practice

The mapping of daily activities showed that recreational behaviour in psychogeriatric nursing homes is strongly shaped by organisational routines, central communal spaces, and staff-led initiatives, confirming earlier work on the structuring role of the institutional environment in dementia care [1,60]. Consistent with prior observational studies [1,41], most activities were either staff-organised group events (for example, bingo or themed games) or dyadic interactions (such as reading the newspaper together or assisted gardening), with only occasional self-initiated individual activities such as knitting or reading being observed. This pattern fits other work that shows how activity options for people with dementia are often organised around staff schedules and institutional priorities rather than spontaneous resident-driven engagement [61], especially in later stages of the condition, such as you find in Dutch nursing homes. The predominance of scheduled and selectively invited group activities, such as the bingo session where only pre-selected residents were asked to join, illustrates how organisational routines can limit access to potentially facilitative social situations for non-invited observers.

Interactions that were observed often clustered around central dining tables and in living rooms, where visibility and proximity increased the likelihood that residents would notice others’ activities [41]. The present study extends this literature by suggesting that not only the occurrence but also the social ripple effects of activities are strongly dependent on where and when they are situated within the organisational rhythms and day-to-day lives of residents with dementia.

The narratives further highlighted that, next to institutional context, social and physical context influence whether activities could become socially ‘contagious’ in the sense implied by response facilitation. Individually performed hobbies such as knitting or reading, when performed at the periphery of the room, tended to remain socially ‘silent’ for other residents, whereas similar activities at the central table occasionally prompted small exchanges or requests to share the material, as seen in the newspaper narratives. This nuance resonates with Social Cognitive Theory’s emphasis on attentional processes: only behaviour that is both visible and contextually salient is likely to function as a model that can support response facilitation [16,62,63].

### 5.3. Scenarios: Behavioural and Physiological Indicators of Engagement Across Social Contexts

Behaviourally, individual activities appeared least facilitative, generating only fleeting glances or material-sharing requests when centrally visible, but even then, rarely sustaining attention or prompting action. This may reflect Social Cognitive Theory’s emphasis on attentional processes: research in other target groups shows that in some cases solitary models lack the salience and social amplification required to elevate behavioural activation [20,62], whereas multiple peers and distributed social cues enhance collective attentional capture [64,65]. Dyadic settings seemed to introduce modest social momentum through caregiver-resident exchanges, eliciting some verbal comments and physical engagement, though participation remained sporadic. Group activities appeared to catalyse relatively more and a broader spectrum of engagement and involvement via distributed cues like shared laughter, object trajectories, caregiver/volunteer social cues (verbal or non-verbal), or peer transitions, potentially creating natural on-ramps for (sometimes reluctant) observers. From an SCT perspective, dyadic contexts may capture partial attention via authority cues (caregivers), but group context can use collective reinforcement [65], social scaffolding [66], and peer-similarity mechanisms [15] that could promote vicarious incentive motivation, also in older adults with moderate to advanced dementia.

Physiologically, patterns seemed to mirror this behavioural hierarchy but with notable sensitivity: individual contexts yielded minimal signals, dyadic elicited covert focus or amusement that appeared decoupled from visible behaviour, and groups showed arousal (attention and excitement) that often seemed to precede or amplify ‘overt’ behaviour. Dyadic physiological activation frequently occurred without behavioural translation and vice versa, which was not the case for individual and group contexts. Earlier [1] and other [67] research also shows that in people with dementia, physiological signals may occur without corresponding overt behaviour, or vice versa. This may be consistent with research showing weak concordance between arousal and overt expression in older and clinical populations [67,68], potentially reflecting motor and sensory inhibition declines distinct from sensory processing [69,70] when social cues remain ambiguous or personal preference resists participation. Future research could explore whether the difference between social context, i.e., the dyadic versus the other two contexts, is due to dyadic settings creating more ambiguity (one authority figure, unclear group norms) that, in turn, only trigger partial engagement (e.g., physiological signs/priming without commitment to action). It could be in line with prior literature on modelling, suggesting that groups are better aligned with the institutional realities (staff-led routines, peer proximity) to generate more engagement [71,72], though multimodal assessment appears necessary to detect these effects.

These patterns may challenge prevailing narratives of apathy as pure disengagement: inactivity might often conceal cognitive or emotional engagement, detectable perhaps only through synchronised observation and physiological data [1,61,73]. Research increasingly distinguishes apathy (motivational deficit) from passivity (motor inertia), with evidence that apathetic residents can engage when external scaffolding reduces effort costs and amplifies reward salience [61,73,74]. Given apathy’s prevalence in nursing home populations, group activities may function as social reward amplifiers for response facilitation: collective reactions, clear structure, and visible peer engagement may help in transforming high-effort tasks into lower-threshold experiences. Future research could further explore whether group-based social rewards selectively enhance response facilitation in residents with high apathy severity, and whether repeated exposure to group modelling gradually lowers the effort threshold for independent initiation.

Across all observations and physiological data measurements, observed reactions predominantly seem to be relaxation, amusement, or cognitive engagement, with no significant indications of stress or negative affect. Despite this, not all interaction is the same; e.g., some residents display silent engagement (e.g., sustained cognitive attention without overt behavioural response), while others may show physiological arousal that does not translate into observable participation/action (see above). It is notable that interactions were almost always experienced positively (or neutrally); however, this positivity may also be influenced by the predominantly positive interactions that were observed and analysed during the research period, warranting careful interpretation when generalising findings. Any negative, stressful interactions that were observed (informally) were taking place in hallways (negative verbal comments during wandering, refusal to go somewhere), in personal rooms (stress during dressing, for example), or in bathrooms (not wanting to be washed).

### 5.4. Conditions That May Influence Response Facilitation

#### 5.4.1. The Role of Social Interaction: Content, Participants, and Accessibility

Observing social interaction alone may not be sufficient to guarantee engagement, as neither spatial proximity nor verbal cues were consistently linked to cognitive involvement. Some residents’ scenarios showed cognitive activation and amusement when observing the activity from a distance, while others are right there at the same table, looking at the activity, and do not (behaviourally or physiologically) react. So while it seems that for some residents, looking at social interactions (without understanding or hearing) may still facilitate engagement or elicit positive responses, for others, the content of interaction and the participants involved may also shape responses. The available data does not permit strong conclusions regarding which specific aspects (who, what topic, how interaction is led) determine engagement and to what extent this is universal or person-specific.

From communication research in dementia care, we know that infantilising tone, rapid pacing, and emotional escalation elicit resistance, whilst calm, patient-centred communication with preserved dignity shows improved engagement [75,76]. Central variables from Bandura’s framework relevant for modelling (from research with other target groups) are, for example, perceived authority, model prestige, familiarity, and emotional expressivity [15,77] and have received little empirical attention in dementia populations, particularly in *indirect* contexts such as response facilitation, wherein the person with dementia observes the caregiver’s communicative model as a bystander rather than intended recipient. Future research should explore these aspects in more detail.

#### 5.4.2. The Influence of Activity Type and Facilitation

The nature of the activity itself seems to play a role in shaping engagement. Activities with dynamic materials (such as balls in table games) may be better able to evoke more immediate and cognitively engaged responses compared to more static materials (like newspapers). That being said, engagement with materials does not necessarily equate to cognitive engagement. Social scaffolding (such as prompts, modelling, or encouragement from staff and peers) frequently appears necessary to bridge the gap between (physically) passive observation and active participation. One reason why residents seem to respond more often to group games may be that centralised, guided activities (i.e., those led by care professionals or volunteers) appear to structure the physical environment and social cues in ways that support more collective engagement, including more opportunities for social scaffolding. This connects to other research suggesting that ‘doing things together’ can be beneficial to how activities are experienced [78] as well as the structured nature of activities that may allow for more and clearer social cues [71].

Not only do materials of the activity play a role, but the expectations, habits, and how the activities are usually done in (past) daily life can be relevant: some hobby activities are more often performed individually or in pairs than others. For example, reading, knitting, making art (sometimes), and puzzles may all be activities that other residents think of as ‘do not disturb’ activities, despite the presence of staff or volunteer facilitation. Residents’ past life experiences with these activities may thus create informal barriers to participation, independent of the physical arrangement of the activity itself [79,80]. Conversely, activities such as group games or collective crafts, which may be culturally associated with communal participation, may more readily invite observation and spontaneous joining, as they perhaps do not carry the same implicit signal of individual activity.

In terms of physical configuration, individual and dyadic activities often feature close positioning between caregivers and residents, with shared attention directed towards a singular object or task. Such configurations, with inward body language/body positioning and (more) exclusive spatial arrangements, may implicitly signal intimacy and restrict accessibility, thus (sometimes unintentionally) creating barriers for wider participation. In contrast, group activities frequently utilise more open physical layouts, with tables arranged for collective participation or ball games allowing for movement (of the ball) between participants, which may foster more inclusiveness and a sense of invitation to onlookers. Research shows the importance of spatiotemporal arrangements for activities in people with dementia [81], but more research should be done to widen the scope from the spatial organisation of objects to the general layout of space and spatial affordances as well.

Lastly, established routines and habitual ways of organising activities within the care environment further reinforce these differences. Established routines that consistently deliver activities in a particular format (individual or dyadic) tend to solidify expectations for both staff and residents, regardless of changes in social context [47,80]. Caregivers may unknowingly perpetuate such delivery modes, overlooking opportunities to adapt the positioning, materials, or their facilitative approach. Introducing variety in spatial arrangements or explicitly inviting participation may thus enhance social accessibility and engagement for other residents in communal settings.

### 5.5. Methodological Considerations and Implications for Future Research

Interpretation of results must acknowledge several limitations. Technical challenges with physiological sensor compliance and data completeness constrained both sample size and the robustness of findings. Naturalistic observation risks both observer and participant reactivity. The narrow diagnostic profile and focus on Dutch nursing home settings may limit generalisability. Furthermore, ambiguity remains surrounding the definition and assessment of “engagement”, where neither behaviour nor physiological measurements alone provide an unequivocal answer [41]. More research into how this physical and/or cognitive engagement is experienced by the person with dementia, as well as how it is exhibited in different types of data (physiological, observational, proxy interviews/interpretation from caregivers), is necessary. The same applies to the opposite: namely, ‘inactivity’ and how it is exhibited and experienced by the older adults with dementia themselves [56]. Future research should further look into the interpretation of engagement and inactivity, combining physiological measurements and observational tools as well as contextual factors.

Additionally, selection bias may have influenced some of the findings. Wards were selected based on practical factors such as staff availability and ethical approval, which could systematically favour locations with more cooperative residents or staff who are positively disposed towards recreational activities. Similarly, informal caregiver consent might have been more readily granted for less challenging residents (e.g., those exhibiting lower levels of agitation, aggression, or apathy), potentially excluding individuals with pronounced behavioural symptoms who could also benefit from response facilitation/participation in activities. While inherent to naturalistic research involving vulnerable populations, this selection process could have yielded a sample that is more amenable to engagement, thereby limiting generalisability to the broader psychogeriatric nursing home population.

Despite these constraints, the multi-method approach (narratives + scenarios + physiology) offers novel insight into response facilitation’s operation. Future research should examine how staff communication style, group composition, activity content, and individual factors (dementia type/severity, neuropsychiatric profile) interact to shape overt/covert engagement, using longitudinal designs to test whether repeated group exposure transforms transient facilitation into more stable participation patterns. The multi-method approach could be enhanced by adding ethnographic approaches as well to even better illuminate residents’ lived experiences of physiological arousal without action or apparent inactivity, thereby refining engagement conceptualisations that respect autonomy while leveraging social mechanisms.

## 6. Conclusions

The main question of this article was, Within which social contexts can response facilitation effectively engage older adults with moderate to advanced dementia in recreational activities in nursing homes? From our research, it seems that group-based social contexts provide the strongest conditions for response facilitation, as non-participating residents displayed more frequent and more consistent behavioural and physiological signs of engagement during structured group activities compared to individual or dyadic settings.

In everyday practice across four psychogeriatric wards, recreational activities were mainly organised as dyadic staff-resident interactions or structured group sessions, with few self-initiated individual activities and with central communal spaces functioning as key arenas for potential modelling and observation. At the same time, observational and physiological indicators often diverged, indicating that residents may experience cognitive or emotional engagement even when their behaviour appears passive or verbally dismissive, highlighting the risk that behaviour-only assessments underestimate internal engagement.

Despite methodological limitations, including selection bias and sensor compliance challenges, the multi-method approach (narratives + scenarios + physiology) offers novel insight into response facilitation’s operation. Theoretically, these findings refine the application of Social Cognitive Theory and vicarious incentive motivation to dementia care by demonstrating that response facilitation may still operate in populations with reduced self-regulatory capacities, provided that social and environmental conditions support attention, visibility, and socially scaffolded opportunities for participation. Methodologically, the study contributes by combining narratives, fly-on-the-wall observations, and synchronised physiological measurements to capture both overt and covert facets of engagement in ecologically valid settings. Practically, the results point to the value of designing everyday care routines and environments that prioritise visible, centrally located group activities during high-occupancy periods, inclusive facilitation practices that allow for peripheral involvement, and multimodal approaches for assessing engagement and inactivity beyond behavioural observation alone. Future research should further disentangle how staff communication styles, activity characteristics, and individual differences interact within group contexts to promote or inhibit response facilitation, thereby informing evidence-based, resource-sensitive strategies that harness social mechanisms for meaningful participation in dementia care.

For now, it looks like reliance on environmental design is insufficient; interventions could integrate tailored social support and remain sensitive to the heterogeneity of cognitive profiles as well as integration into daily patterns and care routines. Future research could pursue technology-enabled products or spaces to extend social cues, experiment with causal designs, and clarify mechanisms across subgroups and dementia stages. Ultimately, a nuanced, multimodal approach that integrates environmental, social, technological, and individual strategies will be essential for fostering meaningful participation and well-being among nursing home residents with dementia.

## Figures and Tables

**Table 1:** Overview of data collection and analysis methods (adapted from: [1]).

Research Step		Data Collection	Data Analysis	Goal
1	Mapping daily activities (Section 3.3)	Fly-on-the-wall observation, unstructured interviews	Observation list, topic list	Insights into who is doing what with whom, in what relationship, in what context, and where. Including background knowledge on residents (3.3)
2	Formulating narratives per case study (Section 3.4)	Results of research step 1	Thematic and Interaction Analysis	Identifying common themes of ‘daily living’ i.r.t. facilitation strategies, to inform scenario development (step 3).
3	Constructing and executing scenarios	Fly-on-the-wall observation and videotaping	Observation list	Insights into behavioural actions during scenario execution.
	while measuring the affective response (Section 3.5)	Observational scales, physiological sensors	MEDLO, OERS, HR, and HRV levels	Insights into affective responses.

**Table 2:** Overview of participants, type of dementia, severity. People with a * are people where the sensor did not yield any useful sensor data in any scenario.

	Pseudonym	Type of Dementia	Severity	Symptoms	Sensory Issues	Mobility
Case Study 1	Alice	AD	Moderate-Severe	Moderate Apathy, Memory and Orientation Problems	glasses	walker
	Bernice	AD	Moderate-Severe	Moderate Apathy, Memory and Orientation Problems	n/a	walker
	Caroline	Mix	Moderate	Apathy, Memory and Orientation Problems, Wandering, Emotional Instability	glasses	unaided
	Douglas *	AD	Moderate-Severe	Severe Apathy, Memory and Orientation Problems	glasses	walker
	Edward	Mix	Moderate	Apathy, Memory Problems	glasses	wheelchair
Case Study 2	Ada *	AD + VD	Moderate	Apathy, Memory and Orientation Problems	n/a	unaided
	Byron	Mix	Moderate	Severe Apathy, Memory and Orientation Problems, Wandering, Aggressiveness	n/a	unaided
	Charlotte	AD	Moderate	Apathy, Memory and Orientation Problems, Paranoia	n/a	walker
	Daisy	AD	Severe	Severe Apathy, Memory and Orientation Problems, Wandering, Impaired communication	n/a	unaided
	Elise	Mix	Severe	Severe Apathy, Memory and Orientation Problems, Severely impaired communication	n/a	wheelchair
Case Study 3	Annie	AD + VD	Moderate-Severe	Severe Apathy, Memory and Orientation Problems, Wandering	glasses	walker
	Ben	AD	Severe	Apathy, Memory and Orientation Problems, Wandering, Aggressiveness	n/a	unaided
	Clara *	AD	Moderate-Severe	Apathy, Memory Problems	glasses	walker
	Dora	AD	Moderate	Apathy, Memory Problems	n/a	unaided
	Edith	VD	Moderate-Severe	Severe Apathy, Memory and Orientation Problems, Hearing Problems	glasses/auditory	walker
	Frank	LB	Severe	Apathy, Memory Problems, Wandering, Impaired communication, Hallucinations	n/a	unaided
Case Study 4	Audrey	AD	Moderate-Severe	Apathy, Memory Problems	glasses	unaided
	Blanche *	AD	Severe	Severe Apathy, Memory and Orientation Problems, Wandering	n/a	unaided
	Constance *	AD	Moderate-severe	Apathy, Memory Problems, Paranoia	n/a	wheelchair
	Delilah *	FTD	Moderate-Severe	Apathy, Memory Problems	glasses	walker
	Eleanor	AD	Moderate-Severe	Severe Apathy, Memory, and Orientation Problems	n/a	unaided

* People with a * are people where the sensor did not yield any useful sensor data in any scenario.

**Table 3:** Overview narratives in a social context.

Narrative	Dementia	Stage	Activity Setting	With Whom?	Physical Context	Activity	Time	Initiative
Douglas	AD	moderate-severe	Individual	n/a	Living room	Reading	14.30	yes
Annie	AD + VD	moderate-severe	Individual	n/a	Living room	Knitting	9.30	yes
Edward	mix	moderate	Dyadic	volunteer	Living room	Walking	10.00	no
Byron	mix	moderate	Dyadic	caregiver	Living room	Reading	10.45	no
Bernice	AD	moderate-severe	Group	caregiver	Activity room	Bingo	10.00	no

**Table 4:** Overview of scenarios, per resident, reporting on behaviour, mood, and physiological measurements per scenario.

Resident	Dementia Type	Stage	Involved in Activity	Potential Respondent to Scenario	Observed Behaviour	Observed Mood ^1^	Physiological Measurements
Alice	AD	Moderate-Severe	n/a	individual	none	contentment/interest	Concentration during small conversation; relaxation and amusement during the rest of the scenario
Bernice	AD	Moderate-Severe	n/a	individual	none	interest/pleasure; no agitation	Concentration observed just before and after conversation with Alice (amusement during) and while watching Caroline fold laundry; relaxation followed after Caroline left.
				dyadic	verbal	interest/pleasure; no agitation	Excitement and pleasure during conversation and while observing the game; concentration noted when verbally engaging, followed by amusement after the game has ended.
Caroline	Mix	Moderate	individual	dyadic	verbal	sad/interest; mild agitation	No physiological response to the game; concentration during observation of social interaction, as well as when leaving the room.
Douglas	AD	Moderate-Severe	dyadic	individual	none	contentment/no agitation	**
Edward	Mix	Moderate	n/a	individual	physical	contentment	Excitement during conversation; relaxation while watching Caroline; mild exertion during folding, followed by post-activity relaxation.
				dyadic	none	contentment; no agitation	Excitement when starting dozing, looking at game show focused but calm concentration; pleasure after.
Ada	AD + VD	Moderate	n/a	individual	none	contentment/no agitation	*
				dyadic- reading	none	contentment/no agitation	*
				dyadic- garden	none	contentment/no agitation	*
Byron	Mix	Moderate	individual	dyadic- puzzle	none	contentment/interest; no agitation	Focus during newspaper reading; amusement when observing the lively puzzle interaction, repeating with the visitor’s arrival, and a chat.
				dyadic- garden	none	contentment/interest; no agitation	Relaxation during rest and sitting; concentration when looking at laughter between Charlotte and the volunteer, and when talking.
				dyadic- reading	none	contentment/interest; no agitation	Excitement when looking around the table, followed by amusement.
Charlotte	AD	Moderate	dyadic- garden	individual	none	sadness/contentment; no agitation	*
				dyadic- reading	none	contentment/interest; no agitation	*The physiological data show concentration and mental engagement when observing the dyadic social interaction, with contentment and relaxation when passively viewing other residents.*
Daisy	AD	Severe	n/a	individual	physical and verbal	pleasure/contentment; no agitation	*
				dyadic- puzzle	none	pleasure/contentment; no agitation	**
				dyadic- garden	none	pleasure/contentment; no agitation	*Data show relaxation and amusement during observation of the gardening activity, with no indicators of exertion despite continuous walking and wandering, suggesting engagement with little cognitive activation.*
Elise	Mix	Severe	n/a	dyadic- reading	none	pleasure/no agitation	**
				group	physical	contentment/pleasure; no agitation	Physiological data shows excitement throughout the activity.
Annie	AD + VD	Moderate-Severe	group- game	individual	none	contentment/no agitation	**
				dyadic	none	contentment/no agitation	**
				group-ball	physical and verbal	contentment/interest/pleasure; some agitation	Initially shows amusement, even when refusing to join the game, followed by contentment during participation.
Ben	AD	Severe	n/a	dyadic	none	contentment/interest; no agitation	*
				group-game	none	contentment/interest; no agitation	Ben’s data show mild physical activity during walking, and relaxation plus positive emotion when seated on his walker, watching the game.
				group-ball	physical and verbal	anxiety/contentment/interest/pleasure; no agitation	*
Clara	AD	Moderate-Severe	group-game	dyadic	none	contentment/interest/pleasure; no agitation	**
			group-ball				
Dora	AD	Moderate	group-game	individual	verbal	interest/contentment; no agitation	*
			group-ball	dyadic	verbal	contentment; no agitation	Initially, the resident shows no reaction to the Christmas card materials. Positive emotion and excitement occur while walking around. Later, amusement during observing verbal interaction is seen.
Edith	VD	Moderate-Severe	group-ball	dyadic	physical and verbal	contentment/interest/pleasure; no agitation	Edith shows cognitive engagement when looking to be dozing/observing other residents with the activity. Relaxation and excitement alternate when looking around and laughing. Amusement during fidgeting with the materials. Cognitive engagement occurs just before and during cleaning up the materials.
			individual				
Frank	LB	Severe	n/a	dyadic	none	contentment; no agitation	Physiological data indicate first excitement when entering the room and then moderate physical exertion throughout the activity, with no change when walking past the table.
				group- game	physical and verbal	contentment/anxiety; mild agitation	*Data show excitement during preparatory activities (tidying pillows), followed by cognitive engagement approximately five minutes before verbal participation in the game, diverging from observed signs of anxiety.*
Audrey	AD	Moderate-Severe	group	individual	none	contentment; no agitation	Audrey shows no specific reaction to Delilah’s individual activity. Values show pleasure, excitement, amusement, and relaxation. Cognitive engagement is only seen during singing.
				dyadic	none	contentment/pleasure; no agitation	Shows concentration during a phone conversation of Eleanor with her daughter, followed by relaxation during lunch
Blanche	AD	Severe	group	individual	none	contentment; no agitation	**
Constance	AD	Moderate-severe	n/a	dyadic	none	contentment; no agitation	*
				group	verbal	contentment; no agitation	*
Delilah	FTD	Moderate-Severe	individual	group	none	contentment; no agitation	**
Eleanor	AD	Moderate-Severe	dyadic	group	verbal	interest/contentment; no agitation	*The physiological data show amusement and positive emotional engagement throughout the card-making activity, persisting during negative verbal expressions, indicating incongruence between stated disinterest and physiological markers of pleasure.*

^1^ = Based on OERS (mood) and MEDLO (mood, agitation) observation scales; ** = no sensor on/took off sensor; * = sensor did not work/no usable data; Italics = used in examples in text.

## Data Availability

The datasets presented in this article are not readily available because the data is part of an ongoing PhD study, and the data is pseudonymized but includes personal, health, and contextual data that cannot be completely anonymized. Requests to access the datasets should be directed to the corresponding author.

## Supplementary Materials

The following supporting information can be downloaded at: https://www.mdpi.com/article/10.3390/healthcare14040539/s1, File S1: Principle of the observation list on daily activities; File S2: Observational scales on mood and agitation.

## Abbreviations

The following abbreviations are used in this manuscript:

SCT	Social Cognitive Theory
VD	Vascular Dementia
FTD	Frontotemporal Dementia
AD	Alzheimer’s Disease
LBD	Lewy Body Dementia
PDD	Parkinson’s Disease Dementia
OERS	Observed Emotion Rating Scale
HR	Heart Rate
HRV	Heart Rate Variability
PR	Pulse Rate
PRV	Pulse Rate Variability
SCL	Skin Conductance Level

## References

[1] Hammink J.H.W., van Buuren L.P.G., Moor J.A., Derks D.A., Mohammadi M. (2024). Evolving Dementia Care: An Explorative Study on the Lived Experience of Older Adults Living with Dementia in Nursing Homes Using Observational and Biometric Sensor Data. Dementia.

[2] Cooper C., Mukadam N., Katona C., Lyketsos C.G., Ames D., Rabins P., Engedal K., de Mendonça Lima C., Blazer D., Teri L. (2012). Systematic Review of the Effectiveness of Non-Pharmacological Interventions to Improve Quality of Life of People with Dementia. Int. Psychogeriatr..

[3] Hulme C., Wright J., Crocker T., Oluboyede Y., House A. (2009). Non-Pharmacological Approaches for Dementia That Informal Carers Might Try or Access: A Systematic Review. Int. J. Geriatr. Psychiatry.

[4] Woods B., Aguirre E., Spector A.E., Orrell M. (2023). Cognitive Stimulation to Improve Cognitive Functioning in People with Dementia. Cochrane Database Syst. Rev..

[5] Sommerlad A., Huntley J.D., Liu K.Y., Gonzalez S.C., Selbaek G., Alladi S., Ames D., Banerjee S., Burns A., Brayne C. (2025). Interventions for People Living with Dementia: Updates from 2024 Lancet Commission. Alzheimers Dement..

[6] Smit D. (2017). Predictors of Activity Involvement in Dementia Care Homes: A Cross-Sectional Study. BMC Geriatr..

[7] McAuliffe L., Fetherstonhaugh D., Rayner J.-A., Clune S. (2024). Having to ‘Go beyond’: Staff Perspectives on Activity Programs for Older People Living in Nursing Homes. J. Aging Stud..

[8] Erlandsson S., Knutsson O., Schön U.-K. (2023). Perceptions of Participation: How Nursing Home Staff and Managers Perceive and Strive for Participation of Older Residents. Eur. J. Social. Work..

[9] Costa E. (2024). Examining the Effectiveness of Interventions to Reduce Discriminatory Behavior at Work: An Attitude Dimension Consistency Perspective. J. Appl. Psychol..

[10] Zhang A.L., Liu S., White B.X., Liu X.C., Durantini M., Chan M.-P.S., Dai W., Zhou Y., Leung M., Ye Q. (2024). Health-Promotion Interventions Targeting Multiple Behaviors: A Meta-Analytic Review of General and Behavior-Specific Processes of Change. Psychol. Bull..

[11] Michie S., van Stralen M.M., West R. (2011). The Behaviour Change Wheel: A New Method for Characterising and Designing Behaviour Change Interventions. Implement. Sci..

[12] Glanz K., Rimer B.K., Viswanath K. (2008). Health Behavior and Health Education: Theory, Research, and Practice.

[13] Glanz K., Bishop D.B. (2010). The Role of Behavioral Science Theory in Development and Implementation of Public Health Interventions. Annu. Rev. Public Health.

[14] Painter J.E., Borba C.P.C., Hynes M., Mays D., Glanz K. (2008). The Use of Theory in Health Behavior Research from 2000 to 2005: A Systematic Review. Ann. Behav. Med..

[15] Bandura A. (1986). Social Foundations of Thought and Action: A Social Cognitive Theory.

[16] Bandura A. (2001). Social Cognitive Theory: An Agentic Perspective. Annu. Rev. Psychol..

[17] Di Lorito C., Bosco A., Pollock K., Harwood R.H., das Nair R., Logan P., Goldberg S., Booth V., Vedhara K., Godfrey M. (2020). External Validation of the ‘PHYT in Dementia’, a Theoretical Model Promoting Physical Activity in People with Dementia. Int. J. Environ. Res. Public Health.

[18] Hammink J.H.W., Moor J.A., Mohammadi M. (2023). Influencing Health Behaviour Using Smart Building Interventions for People with Dementia and Mild Cognitive Impairment: Expert Interviews and a Systematic Literature Review. Disabil. Rehabil. Assist. Technol..

[19] Schunk D.H., Usher E.L. (2012). Social Cognitive Theory and Motivation. The Oxford Handbook of Human Motivation.

[20] Bandura A. (1989). Human Agency in Social Cognitive Theory. Am. Psychol..

[21] Jonker C., Slaets J.P.J., Verhey F.R.J. (2009). Handboek Dementie: Laatste Inzichten in Diagnostiek En Behandeling.

[22] Schunk D.H., DiBenedetto M.K. (2020). Motivation and Social Cognitive Theory. Contemp. Educ. Psychol..

[23] Jao Y.-L., Algase D.L., Specht J.K., Williams K. (2016). Developing the Person–Environment Apathy Rating for Persons with Dementia. Aging Ment. Health.

[24] Jao Y.-L., Liu W., Williams K., Chaudhury H., Parajuli J. (2019). Association between Environmental Stimulation and Apathy in Nursing Home Residents with Dementia. Int. Psychogeriatr..

[25] Trahan M.A., Kuo J., Carlson M.C., Gitlin L.N. (2014). A Systematic Review of Strategies to Foster Activity Engagement in Persons With Dementia. Health Educ. Behav..

[26] Cooper L.D., Rigrodsky S. (1979). Verbal Training to Improve Explanations of Conservation with Aphasic Adults. J. Speech Lang. Hear. Res..

[27] Clare L., Jones R.S.P. (2008). Errorless Learning in the Rehabilitation of Memory Impairment: A Critical Review. Neuropsychol. Rev..

[28] Dijksterhuis A., Bargh J.A. (2001). The Perception-Behavior Expressway: Automatic Effects of Social Perception on Social Behavior. Advances in Experimental Social Psychology.

[29] Brass M., Derrfuss J., Matthes-von Cramon G., Von Cramon D.Y. (2003). Imitative Response Tendencies in Patients with Frontal Brain Lesions. Neuropsychology.

[30] Galik E.M., Resnick B., Pretzer-Aboff I. (2009). ‘Knowing What Makes Them Tick’: Motivating Cognitively Impaired Older Adults to Participate in Restorative Care. Int. J. Nurs. Pract..

[31] Galik E.M., Resnick B. (2007). Restorative Care With Cognitively Impaired Older Adults: Moving Beyond Behavior. Top. Geriatr. Rehabil..

[32] Heacock P.R., Beck C.M., Souder E., Mercer S. (1997). Assessing Dressing Ability in Dementia. Geriatr. Nurs..

[33] Ries J.D. (2022). A Framework for Rehabilitation for Older Adults Living with Dementia. Arch. Physiother..

[34] Tsai P.-F., Kitch S., Beck C., Jakobs T., Rettiganti M., Jordan K., Jakobs E., Adair S. (2018). Using an Interactive Video Simulator to Improve Certified Nursing Assistants’ Dressing Assistance and Nursing Home Residents’ Dressing Performance: A Pilot Study. Comput. Inform. Nurs..

[35] Turner J., Mathews R.M., Madden G.J., Dube W.V., Hackenberg T.D., Hanley G.P., Lattal K.A. (2013). Behavioral Gerontology. APA Handbook of Behavior Analysis, Vol. 2: Translating Principles Into Practice.

[36] Barrick A.L., Rader J., Hoeffer B., Sloane P.D., Biddle S. (2008). Bathing Without a Battle: Person-Directed Care of Individuals with Dementia.

[37] Beck C., Heacock P., Mercer S.O., Walls R.C., Rapp C.G., Vogelpohl T.S. (1997). Improving Dressing Behavior in Cognitively Impaired Nursing Home Residents. Nurs. Res..

[38] Cruz J., Marques A., Barbosa A.L., Figueiredo D., Sousa L. (2011). Effects of a Motor and Multisensory-Based Approach on Residents with Moderate-to-Severe Dementia. Am. J. Alzheimers Dis. Other Dement..

[39] Downs A.F.D., Rosenthal T.L., Lichstein K.L. (1988). Modeling Therapies Reduce Avoidance of Bath-Time by the Institutionalized Elderly. Behav. Ther..

[40] Boothby E.J., Clark M.S., Bargh J.A. (2014). Shared Experiences Are Amplified. Psychol. Sci..

[41] Cohen-Mansfield J., Thein K., Dakheel-Ali M., Marx M.S. (2010). Engaging Nursing Home Residents with Dementia in Activities: The Effects of Modeling, Presentation Order, Time of Day, and Setting Characteristics. Aging Ment. Health.

[42] Nakata R., Kawai N. (2017). The “Social” Facilitation of Eating without the Presence of Others: Self-Reflection on Eating Makes Food Taste Better and People Eat More. Physiol. Behav..

[43] Aalten P., Verhey F.R., Boziki M., Bullock R., Byrne E.J., Camus V., Caputo M., Collins D., De Deyn P.P., Elina K. (2007). Neuropsychiatric Syndromes in Dementia. Results from the European Alzheimer Disease Consortium: Part I. Dement. Geriatr. Cogn. Disord..

[44] Akyol M.A., Kucukguclu O., Yener G. (2018). Investigation of Factors Affecting Apathy in Three Major Types of Dementia. Arch. Neuropsychiatry.

[45] Ballard C., Gauthier S., Corbett A., Brayne C., Aarsland D., Jones E. (2011). Alzheimer’s Disease. Lancet.

[46] Halek M., Reuther S., Müller-Widmer R., Trutschel D., Holle D. (2020). Dealing with the Behaviour of Residents with Dementia That Challenges: A Stepped-Wedge Cluster Randomized Trial of Two Types of Dementia-Specific Case Conferences in Nursing Homes (FallDem). Int. J. Nurs. Stud..

[47] Regier N.G., Hodgson N.A., Gitlin L.N. (2017). Characteristics of Activities for Persons With Dementia at the Mild, Moderate, and Severe Stages. Gerontologist.

[48] Roberts E. (2023). A Conversation About the Ethics of Past and Future Memory Care Models: Perspectives from the First Two European Dementia Villages. Inquiry.

[49] Connelly F.M., Clandinin D.J. (1990). Stories of Experience and Narrative Inquiry. Educ. Res..

[50] Grimaldi S., Fokkinga S., Ocnarescu I. (2013). Narratives in Design: A Study of the Types, Applications and Functions of Narratives in Design Practice. Proceedings of the 6th International Conference on Designing Pleasurable Products and Interfaces—DPPI ’13.

[51] Mohammadi M., Dominicus M., van Buuren L., Hamers K., Hammink C., Yegenoglu H. (2019). The Evolution of Housing Typologies for Older Adults in The Netherlands From 1945 to 2016: An Analysis in the Context of Policy, Societal, and Technological Developments. J. Hous. Elder..

[52] Emrani S., Lamar M., Price C.C., Wasserman V., Matusz E.F., Au R., Swenson R., Nagele R.G., Heilman K.M., Libon D.J. (2019). Alzheimer’s/Vascular Spectrum Dementia: Classification in Addition to Diagnosis. J. Alzheimer’s Dis..

[53] den Ouden M., Bleijlevens M.H.C., Meijers J.M.M., Zwakhalen S.M.G., Braun S.M., Tan F.E.S., Hamers J.P.H. (2015). Daily (In)Activities of Nursing Home Residents in Their Wards: An Observation Study. J. Am. Med. Dir. Assoc..

[54] Shephard R.J. (2003). Limits to the Measurement of Habitual Physical Activity by Questionnaires * Commentary. Br. J. Sports Med..

[55] Zeisel J. (1993). Inquiry by Design: Tools for Environment-Behaviour Research.

[56] van Buuren L., Hammink C., Mohammadi M., Derks D., Moor N. Constructing the Lived Experience of Older Adults with Dementia: Lessons Learned from an Explorative Mixed Method Approach. Proceedings of the SHE2024 International Scientific Conference.

[57] van Buuren L.P.G. (2019). Slim Wonen Ontzorgt: Ontwerpprincipes En Randvoorwaarden Voor Een Slimme Intramurale Woonzorgomgeving. PDEng Thesis.

[58] Driessen A. (2019). A Good Life with Dementia: Ethnographic Articulations of Everyday Life and Care in Dutch Nursing Homes.

[59] Hirschauer S. (2006). Puttings Things into Words. Ethnographic Description and the Silence of the Social. Human. Stud..

[60] de Boer B., Beerens H.C., Zwakhalen S.M.G., Tan F.E.S., Hamers J.P.H., Verbeek H. (2016). Daily Lives of Residents with Dementia in Nursing Homes: Development of the Maastricht Electronic Daily Life Observation Tool. Int. Psychogeriatr..

[61] Leone E., Deudon A., Piano J., Robert P., Dechamps A. (2012). Are Dementia Patient’s Engagement Using Tailored Stimuli the Same? The Apathy Dilemma in Nursing Home Residents. Curr. Gerontol. Geriatr. Res..

[62] Rosenthal T., Bandura A., Garfield S., Bergin A. (1978). Handbook of Psychotherapy and Behavior Change.

[63] Luszczynska A., Schwarzer R. (2005). Social Cognitive Theory. Predict. Health Behav..

[64] Vollet J.W., Kindermann T.A., Skinner E.A. (2017). In Peer Matters, Teachers Matter: Peer Group Influences on Students’ Engagement Depend on Teacher Involvement. J. Educ. Psychol..

[65] Shteynberg G., Hirsh J.B., Bentley R.A., Garthoff J. (2020). Shared Worlds and Shared Minds: A Theory of Collective Learning and a Psychology of Common Knowledge. Psychol. Rev..

[66] Kraatz E., Nagpal M., Lin T.-J., Hsieh M.-Y., Ha S.Y., Kim S., Shin S. (2020). Teacher Scaffolding of Social and Intellectual Collaboration in Small Groups: A Comparative Case Study. Front. Psychol..

[67] Steinert L., Putze F., Küster D., Schultz T. (2022). Predicting Activation Liking of People With Dementia. Front. Comput. Sci..

[68] Zantige G., van Rijn S., Stockmann L., Swaab H. (2019). Concordance between Physiological Arousal and Emotion Expression during Fear in Young Children with Autism Spectrum Disorders. Autism.

[69] Healey R., Goldsworthy M., Salomoni S., Weber S., Kemp S., Hinder M.R., St George R.J. (2024). Impaired Motor Inhibition during Perceptual Inhibition in Older, but Not Younger Adults: A Psychophysiological Study. Sci. Rep..

[70] Persad C.C., Abeles N., Zacks R.T., Denburg N.L. (2002). Inhibitory Changes After Age 60 and Their Relationship to Measures of Attention and Memory. J. Gerontol. B Psychol. Sci. Soc. Sci..

[71] Cohen-Mansfield J. (2018). The Impact of Group Activities and Their Content on Persons with Dementia Attending Them. Alzheimers Res. Ther..

[72] Jesdale B.M., Bova C.A., Mbrah A.K., Lapane K.L. (2021). Group Activity Participation in Relation to Contextual Isolation of United States Nursing Home Residents Living with Alzheimer’s Disease and Related Dementias. J. Nurs. Home Res. Sci..

[73] Wong S., Wei G., Husain M., Hodges J.R., Piguet O., Irish M., Kumfor F. (2023). Altered Reward Processing Underpins Emotional Apathy in Dementia. Cogn. Affect. Behav. Neurosci..

[74] Attaallah B., Toniolo S., Maio M.R., Husain M. (2024). Apathy and Effort-based Decision-making in Alzheimer’s Disease and Subjective Cognitive Impairment. Alzheimer’s Dement. Diagn. Assess. Dis. Monit..

[75] Coleman D.J., Asiri N.A.S. (2020). A Person-Centred Communication Approach to Working with Older People Who Have Dementia. Br. J. Healthc. Assist..

[76] Williams K.N., Perkhounkova Y., Herman R., Bossen A. (2017). A Communication Intervention to Reduce Resistiveness in Dementia Care: A Cluster Randomized Controlled Trial. Gerontologist.

[77] Manz C.C., Sims H.P. (1981). Vicarious Learning: The Influence of Modeling on Organizational Behavior. Acad. Manag. Rev..

[78] McCabe L., Robertson J., Kelly F. (2018). Scaffolding and Working Together: A Qualitative Exploration of Strategies for Everyday Life with Dementia. Age Ageing.

[79] Kolanowski A., Buettner L., Litaker M., Yu F. (2006). Factors That Relate to Activity Engagement in Nursing Home Residents. Am. J. Alzheimers Dis. Other Dement..

[80] Tak S.H., Kedia S., Tongumpun T.M., Hong S.H. (2015). Activity Engagement: Perspectives from Nursing Home Residents with Dementia. Educ. Gerontol..

[81] Majlesi A.R., Ekström A., Hydén L.-C. (2019). Spatiotemporal Arrangement of Objects in Activities with People with Dementia. Logop. Phoniatr. Vocol.

