# Supplementary material for: Response Facilitation in Dementia Care: Exploring Engagement Through Social Contexts: A Qualitative Study in Dutch Nursing Homes
Source: Healthcare (Basel). 2026 Feb 22;14(4):539. doi: 10.3390/healthcare14040539 (PMC12940554; doi:10.3390/healthcare14040539)
Supplement: Supplementary file 1 [file healthcare-14-00539-s001.zip › healthcare-4081960-supplementary.pdf]

## Supplementary file S1

*Supplementary file S1.A Principle of the observation list on daily activities*

**DATE:** **LOCATION:**

| nr[1] | time | who[2] | what[3] | With whom[4] | relationship[5] | remarks[6] |
|---|---|---|---|---|---|---|
| 1 | | | | | | |

[1] **Where**. Where an activity took place is mapped and annotated on the floorplan of the ward. However, not only aspects visible on floorplans are relevant, also environmental conditions may influence the behavior during the activity (Zeisel, 1993; p163). During the observations, not only markings of the floorplan of where an activity took place, also notes regarding the weather conditions, indoor climate, and noise were made. Furthermore, conditions that can be seen as 'barriers' (Zeisel, 1993; p132) were indicated as well. These barriers include walls, screens, objects (such as an open or closed door), and symbols. These environmental conditions are particularly relevant for people with dementia due to their increased sensitivity towards them

[2] **Who**. In order to be able to interpret the activities of people with dementia in real-life settings, it is important to obtain a complete image of personal abilities, preferences, and histories per observed person (i.e., personal context). Dementia can impact cognitive, social, and physical functioning. Examples of physical functioning are whether people were able to walk (unassisted), had visual or auditive impairments, or had impaired motor abilities (Reisberg, et al., 1982). Before the observations started, information about personal background and social functioning was obtained via unstructured interviews with staff.

[3] **What**. For the list of potential observed activities, findings from different studies were combined (den Ouden, et al., 2015; Nordin, et al., 2017; van Buuren, et al., 2019). Of this extensive list of activities, only activities that were taking place (or could take place) in communal areas were included. Activities taking place in private bedrooms and bathrooms (e.g., showering, dressing) where therefore excluded from the list. The list was consequently discussed with staff of the nursing home, in order to check if items were missing.

[4] **With whom**. For the observation list, not only what people were doing was relevant, also with whom. If there was interaction with other residents in the ward, this can be indicated using the abbreviations as described in 'who'. Furthermore, staff that is working on the day of the observations are also inventoried and given a distinctive code for observations (S1, S2, …) Lastly, other people, such as visitors/family were noted and indicated if relevant/occurring during the observations. Descriptions of people were noted and checked with nursing staff or resident afterwards.

[5] **Relationship**. In this element, the connections between 'who' and 'with whom' were described per activity. Descriptions go beyond 'doing together' or 'independently' (Zeisel, 1993), by describing the exact interaction between the resident with dementia and staff/visitor/other. Specific attention was paid to physical actions and (non)verbal interaction when doing an activity; these actions or interactions were noted down.

[6] **Remarks**. The context in which certain activities took place, was highly relevant for observers; it may mean a difference between 'normal' behavior during the activity and behavior that was considered 'disruptive' or 'annoying' (Zeisel, 1993). Therefore, additional notes on personal, social, organizational, spatial, digital, and temporal context was noted down in the remark-column..

| | | | | | | |
|---|---|---|---|---|---|---|
| *n* | | | | | | |

*Supplementary file S1.B Observation list on daily activities category 'what'*

| Category | Subcategory | Activities |
|---|---|---|
| **Inactivity** | **No activity** | Doing 'nothing' |
| | **Watching TV** | Watching TV |
| | **Sleeping** | Sleeping |
| **Activities of Daily Life (ADL)** | **Personal hygiene** | Personal hygiene (e.g., washing hands) |
| | | Put on glasses |
| | | Put on hearing aid |
| | | Taking drugs |
| | | Wipe hands |
| | **Go to bathroom** | Go to bathroom |
| | **Eating and drinking** | Eating |
| | | Drinking |
| | **Mobility** | Physiotherapy |
| | | Transfer from point A to point B |
| | | Looking at info signs and landmarks (for wayfinding purposes) |
| | | Pronouncing aloud destination (for wayfinding purposes) |
| | | Trying to open doors (for wayfinding purposes) |
| | | Help of care professional / informal carer / fellow resident (e.g., physical help, providing directions) (for wayfinding purposes) |
| | | Arrival at destination (for wayfinding purposes) |
| | | Traveled route (for wayfinding purposes) |
| | | Stops on the route (for wayfinding purposes) |
| **Instrumental Activities of Daily Life** | **Domestic activities** | Setting the table |
| | | Cleaning (e.g., sweeping, washing/drying dishes, dusting) |
| | **Preparing food and drinks** | (Helping with) preparing food (e.g., washing, peeling, cutting) |
| | | Stir into drink with a spoon |
| **Communication & hobbies** | **Communication** | Verbal communication |
| | | Non-verbal communication |
| | | Visit |
| | | Phone call (including video calls) |
| | **Hobbies** | Individual activities (e.g., reading, crafting, looking at pictures, etc.) |
| | | Organized activities (e.g., sporting at the table, making music, gardening, playing games) |
| **Remaining** | - | - |

## Supplementary File S2 Observational scales on mood and agitation

*Supplementary File S2.A Observed Emotion Rating Scale (OERS) [55]*

| Category | Signs |
|---|---|
| Pleasure | Smile, laugh, stroking, touching with "approach" manner, nodding, singing, arm or hand outreach, open-arm gesture, eye crinkled |
| Anger | Clench teeth, grimace, shout, curse, berate, push, physical aggression or implied aggression, like fist shaking, pursed lips, eyes narrowed, knit brows/lowered |
| Anxiety/fear | Furrowed brow, motoric restlessness, repeated or agitated motions, facial expression of fear or worry, sigh, withdraw from other, tremor, tight facial muscles, calls repetitively, hand wringing, leg jiggling, eyes wide |
| Sadness | Cry, tears, moan, mouth turned down at corners, eyes/head down turned and face expressionless, wiping eyes, horse-shoe on forehead |

| | |
|---|---|
| Interest | Eyes follow object, intent fixation on object or person, visual scanning, facial, motoric or verbal feedback to other, eye contact maintained, body or vocal response to music, wide angle subtended by gaze, turn body or move toward person or object |
| Contentment | Comfortable posture, sitting or lying down, smooth facial muscles, lack of tension in limbs, neck, slow movements |

*Supplementary file S2.B Maastricht Electronic Daily Life Observation Tool (MEDLO) [56]*

| | Descriptive expressions |
|---|---|
| Negative: a negative mood can be characterized by groaning, moaning, crying, screaming, shouting, tensed facial expression or tensed body language. Furthermore, the content and tone of the verbal or nonverbal interactions gives information regarding the negativity of the mood. | |
| 1 | Great signs of negative mood (sadness, displeasure, anger, worries, fear, boredom or discomfort). |
| 2 | Considerable signs of negative mood (sadness, displeasure, anger, worries, fear, boredom or discomfort). |
| 3 | Small signs of negative mood (sadness, displeasure, anger, worries, fear, boredom or discomfort). |
| 4 | Neutral: a neutral mood is scored if there is no positive or negative mood observable, e.g. gazing or sleeping. |
| Positive: a positive mood can be characterized by smiling, laughing, chuckling, humming a tune, relaxed facial expression or relaxed body language. Furthermore, the content and tone of the verbal or nonverbal interaction gives information regarding the positivity of the mood. | |
| 5 | Small signs of positive mood (contentment, happiness, pleasure, relaxation, comfort). |
| 6 | Considerable signs of positive mood (contentment, happiness, pleasure, relaxation, comfort). |
| 7 | Great signs of positive mood (contentment, happiness, pleasure, relaxation, comfort). |

*Supplementary file S2.C Observational scale on agitation (MEDLO) [56]*

| | Deviating verbal expressions | Motoric agitation | Aggressiveness | Resistance to care (professional) |
|---|---|---|---|---|
| 0 | Not present | Not present | Not present | Not present |
| 1 | Low volume, not disruptive in milieu, including crying | Pacing or moving about in chair at normal rate (appears to be seeking comfort, looking for spouse, purposeless movements) | Verbal threats | Procrastination or avoidance |
| 2 | Louder than conversational, mildly disruptive, redirectable | Increased rate of movements, mildly intrusive, easily redirectable | Threatening gestures; no attempt to strike | Verbal/ gesture of refusal |
| 3 | Loud, disruptive, difficult to redirect | Rapid movements, moderately intrusive or disruptive, difficult to redirect | Physical toward property | Pushing away to avoid task |
| 4 | Extremely loud screaming or yelling, highly disruptive, unable to redirect | Intense movements, extremely intrusive or disruptive, not redirectable verbally | Physical toward self or others | Striking out at caregiver |
